# Cellular proteome datasets of human endothelial cells under physiologic state and after treatment with caffeine and epigallocatechin-3-gallate

**DOI:** 10.1016/j.dib.2019.104292

**Published:** 2019-07-22

**Authors:** Chanettee Chanthick, Visith Thongboonkerd

**Affiliations:** Medical Proteomics Unit, Office for Research and Development, Faculty of Medicine Siriraj Hospital, Mahidol University, Bangkok 10700, Thailand

**Keywords:** Cellular response, Coffee, Epigallocatechin-3-gallate, Polyphenol, Proteome, Tea

## Abstract

Human endothelial cells play several significant roles in vascular biology and homeostasis. We report herein cellular proteome datasets of EA.hy926 human endothelial cells under physiologic condition and after treatment with 100 μM caffeine or EGCG for 24-h. Cellular proteins were extracted and subjected to in-solution tryptic digestion using filter-aided sample preparation (FASP) method. The digested peptides were analyzed by nanoflow liquid chromatography coupled to tandem mass spectrometry (nanoLC-ESI-Qq-TOF MS/MS). Finally, the mass spectral data were searched against the human Swiss-Prot database using Mascot 2.4 search engine and quantified using Skyline v.3.5 software and BiblioSpec algorithm. All of these data were used for further comparative proteomics study followed by bioinformatics analyses to investigate differential biochemical effects of caffeine and EGCG on human endothelial cells (Chanthick et al., 2019) [1].

**Table:** Specifications Table

Subject	Cell biology
Specific subject area	Human endothelial cell proteome
Type of data	Table
How data were acquired	Mass spectrometry, EASY-nLC II (Bruker Daltonics; Bremen, Germany), Qq-TOF MS/MS system (maXis Impact) (Bruker Daltonics), Mascot 2.4 search engine (Matrix Science; London, UK)
Data format	Raw and Analyzed
Parameters for data collection	EA.hy926 human endothelial cells were treated with 100 μM caffeine or EGCG for 24-h, whereas the untreated cells served as the control. Thereafter, cellular proteins were extracted and subjected to gel-free proteome analysis.
Description of data collection	Cellular proteins were subjected to in-solution tryptic digestion and analyzed by nanoLC-ESI-Qq-TOF MS/MS. The mass spectral data were searched against the human Swiss-Prot database using Mascot 2.4 search engine. Fixed modification was carbamidomethylation at cysteine residues, whereas variable modification was oxidation at methionine residues. Enzyme was specified to trypsin and only one missed cleavage per peptide was allowed. Data searches were performed with a precursor tolerance of 0.1 Da and fragmentation tolerance of 0.5 Da with +2 and +3 charge state. The false discovery rate (FDR) was performed by searching the decoy database and adjusted to <1% at protein level.
Data source location	Medical Proteomics Unit, Office for Research and Development, Faculty of Medicine Siriraj Hospital, Mahidol University, Bangkok 10700, Thailand
Data accessibility	With the article.
Related research article	Author's name: Chanettee Chanthick and Visith Thongboonkerd Title: Comparative proteomics reveals concordant and discordant biochemical effects of caffeine versus epigallocatechin-3-gallate in human endothelial cells. Journal: Toxicology and Applied Pharmacology https://doi.org/10.1016/j.taap.2019.114621

**Table:** 

**Value of the Data** • These data are the valuable resource for understanding and further analyzing the cellular proteome of human endothelial cells under physiologic condition and after treatment with caffeine or EGCG. • Proteomists, biochemists, physiologists, cellular biologists, and nutritionists will be benefited from these data. • Further functional investigations on these data will lead to better understanding and provide significant insights into vascular cell biology.

## 1 Data

We report herein the complete lists of proteins identified from EA.hy926 human endothelial cells under physiologic (control) condition (Table 1), after 24-h treatment with caffeine (Table 2), and after 24-h treatment with EGCG (Table 3). Their identities, gene symbols, MS/MS identification scores, percentages of sequence coverage, numbers of distinct and total matched peptides, isoelectric points (p*I*), and intensity data are shown. All of these data were used for further comparative proteomics study followed by bioinformatics analyses to investigate differential biochemical effects of caffeine and EGCG on human endothelial cells [1].

**Table 1:** All proteins identified in control EA.hy926 endothelial cells.

Protein name	Swiss-Prot ID	Gene symbol	MS/MS identification score	% Cov	No. of distinct/total matched peptides	p*I*	Intensity (x10^3^ arbitrary unit)				
							N1	N2	N3	Mean	SEM
10 kDa heat shock protein, mitochondrial	P61604	*HSPE1*	44	29.4	3/5	8.9	17446	3953	17914	13104	4578
14-3-3 protein beta/alpha	P31946	*YWHAB*	147	20.7	4/7	4.8	62742	40199	33195	45379	8914
14-3-3 protein epsilon	P62258	*YWHAE*	136	16.5	3/7	4.6	10727	16190	14735	13884	1634
14-3-3 protein theta	P27348	*YWHAQ*	80	9.4	2/2	4.7	29078	40931	31645	33885	3600
14-3-3 protein zeta/delta	P63104	*YWHAZ*	114	41.2	8/19	4.7	16087	17116	17650	16951	459
26S protease regulatory subunit 6A	P17980	*PSMC3*	70	7.7	2/6	5.1	34193	44909	32290	37131	3928
26S protease regulatory subunit 7	P35998	*PSMC2*	75	3.5	1/2	5.7	10881	24215	19907	18334	3929
26S proteasome non-ATPase regulatory subunit 12	O00232	*PSMD12*	31	8.3	3/4	7.5	19198	46089	29657	31648	7826
3-ketoacyl-CoA thiolase, mitochondrial	P42765	*ACAA2*	41	6.5	1/2	8.3	NA	NA	NA	NA	NA
40S ribosomal protein S10	P46783	*RPS10*	36	14.5	2/2	10.2	NA	NA	NA	NA	NA
40S ribosomal protein S11	P62280	*RPS11*	64	19.6	3/5	10.3	3489	2649	2148	2762	391
40S ribosomal protein S12	P25398	*RPS12*	123	28	3/7	6.8	5989	9223	23754	12989	5463
40S ribosomal protein S13	P62277	*RPS13*	147	18.5	3/7	10.5	4685	6514	5082	5427	555
40S ribosomal protein S14	P62263	*RPS14*	123	7.3	1/2	10.1	12172	18893	13314	14793	2076
40S ribosomal protein S15	P62841	*RPS15*	130	36.6	3/5	10.4	3375	3785	7763	4974	1399
40S ribosomal protein S16	P62249	*RPS16*	208	21.9	3/6	10.2	18328	37786	15543	23886	6996
40S ribosomal protein S18	P62269	*RPS18*	44	8.6	2/4	11.0	3673	3509	3717	3633	63
40S ribosomal protein S19	P39019	*RPS19*	61	21.4	3/5	10.3	31871	48586	40790	40416	4829
40S ribosomal protein S2	P15880	*RPS2*	99	17.1	3/6	10.3	23414	25333	32355	27034	2718
40S ribosomal protein S20	P60866	*RPS20*	81	10.1	1/4	10.0	15479	11566	10693	12579	1471
40S ribosomal protein S25	P62851	*RPS25*	57	19.2	2/3	10.1	3224	2039	1350	2204	547
40S ribosomal protein S28	P62857	*RPS28*	49	34.8	2/2	10.7	3816	4070	7180	5022	1082
40S ribosomal protein S3	P23396	*RPS3*	57	10.7	2/4	9.7	5415	10341	6694	7483	1476
40S ribosomal protein S4, X isoform	P62701	*RPS4X*	37	15.6	3/3	10.2	4423	8517	5820	6253	1202
40S ribosomal protein S5	P46782	*RPS5*	763	17.6	4/21	9.7	81783	95839	67353	81658	8223
40S ribosomal protein S6	P62753	*RPS6*	58	13.3	3/6	10.9	3259	26696	7849	12601	7171
40S ribosomal protein S7	P62081	*RPS7*	139	19.6	3/7	10.1	3898	8383	5379	5887	1319
40S ribosomal protein S8	P62241	*RPS8*	61	10.6	2/4	10.3	4996	7677	6009	6227	782
40S ribosomal protein SA	P08865	*RPSA*	98	18.6	3/6	4.8	6334	13254	5954	8514	2372
60 kDa heat shock protein, mitochondrial	P10809	*HSPD1*	783	23	9/27	5.7	36973	86169	86629	69923	16476
60S acidic ribosomal protein P0-like	Q8NHW5	*RPLP0P6*	78	14.2	3/6	5.4	NA	NA	NA	NA	NA
60S acidic ribosomal protein P1	P05386	*RPLP1*	38	14	1/2	4.3	26901	51602	38425	38976	7136
60S acidic ribosomal protein P2	P05387	*RPLP2*	75	10.4	1/4	4.4	17980	10622	14169	14257	2124
60S ribosomal protein L10	P27635	*RPL10*	134	16.4	3/4	10.1	NA	NA	NA	NA	NA
60S ribosomal protein L10a	P62906	*RPL10A*	45	20.7	4/5	9.9	25064	23888	22812	23922	650
60S ribosomal protein L12	P30050	*RPL12*	121	37.6	4/6	9.5	7612	6403	17450	10489	3498
60S ribosomal protein L13	P26373	*RPL13*	30	4.7	1/2	11.7	9680	15437	6803	10640	2538
60S ribosomal protein L18	Q07020	*RPL18*	106	14.4	2/5	11.7	8748	4197	6877	6607	1321
60S ribosomal protein L19	P84098	*RPL19*	102	13.3	2/4	11.5	87869	45599	89801	74423	14423
60S ribosomal protein L23	P62829	*RPL23*	118	25	2/3	10.5	20056	10519	13973	14850	2788
60S ribosomal protein L23a	P62750	*RPL23A*	49	13.5	2/4	10.4	5572	12897	10821	9763	2180
60S ribosomal protein L27	P61353	*RPL27*	31	6.6	1/2	10.6	16193	1895	17547	11878	5007
60S ribosomal protein L30	P62888	*RPL30*	84	13.9	1/2	9.7	5889	37749	46669	30103	12377
60S ribosomal protein L31	P62899	*RPL31*	25	18.4	1/1	10.5	NA	NA	NA	NA	NA
60S ribosomal protein L38	P63173	*RPL38*	95	35.7	2/8	10.1	NA	NA	NA	NA	NA
60S ribosomal protein L6	Q02878	*RPL6*	30	8.7	3/4	10.6	4941	4573	6616	5377	629
6-phosphogluconate dehydrogenase, decarboxylating	P52209	*PGD*	27	2.3	1/10	6.8	NA	NA	NA	NA	NA
78 kDa glucose-regulated protein	P11021	*HSPA5*	903	29.4	18/34	5.1	NA	NA	NA	NA	NA
Acidic leucine-rich nuclear phosphoprotein 32 family member A	P39687	*ANP32A*	71	8.4	1/4	4.0	NA	NA	NA	NA	NA
Actin, aortic smooth muscle	P62736	*ACTA2*	1852	31	11/206	5.2	NA	NA	NA	NA	NA
Actin, cytoplasmic 1	P60709	*ACTB*	17607	50.1	19/1313	5.3	1295615	1587821	1591547	1491661	98029
Activator of 90 kDa heat shock protein ATPase homolog 1	O95433	*AHSA1*	92	9.5	2/3	5.4	NA	NA	NA	NA	NA
Adenosylhomocysteinase	P23526	*AHCY*	35	9.7	3/4	5.9	17980	10622	14169	14257	2124
ADP/ATP translocase 2	P05141	*SLC25A5*	51	10.1	3/7	9.7	33303	52811	47782	44632	5848
ADP-ribosylation factor 1	P84077	*ARF1*	36	17.7	3/3	6.3	NA	NA	NA	NA	NA
Alpha-actinin-1	P12814	*ACTN1*	127	9.5	6/8	5.3	NA	NA	NA	NA	NA
Alpha-actinin-4	O43707	*ACTN4*	306	11.3	7/10	5.3	33344	98134	98063	76514	21585
Alpha-enolase	P06733	*ENO1*	1683	48.4	14/297	7.0	161849	284700	224616	223722	35467
Annexin A1	P04083	*ANXA1*	937	39	10/31	6.6	115245	191854	155100	154066	22121
Annexin A2	P07355	*ANXA2*	1995	57.8	18/64	7.6	NA	NA	NA	NA	NA
Annexin A4	P09525	*ANXA4*	40	10.3	3/3	5.8	32921	44832	30964	36239	4334
Annexin A5	P08758	*ANXA5*	1269	38.8	12/41	4.9	211017	359829	316220	295689	44168
ATP synthase subunit alpha, mitochondrial	P25705	*ATP5F1A*	395	7.4	3/9	9.2	44592	132605	138453	105217	30359
ATP synthase subunit beta, mitochondrial	P06576	*ATP5F1B*	446	27	10/54	5.3	29778	38480	27963	32074	3246
ATP-dependent RNA helicase A	Q08211	*DHX9*	52	3.6	4/9	6.4	6967	4726	3528	5074	1008
ATP-dependent RNA helicase DDX1	Q92499	*DDX1*	69	2.8	1/2	6.8	NA	NA	NA	NA	NA
Barrier-to-autointegration factor	O75531	*BANF1*	114	29.2	2/3	5.8	NA	NA	NA	NA	NA
Basic leucine zipper and W2 domain-containing protein 1	Q7L1Q6	*BZW1*	163	9.8	3/4	5.8	NA	NA	NA	NA	NA
Beta-enolase	P13929	*ENO3*	1090	26.5	6/134	7.6	NA	NA	NA	NA	NA
Calnexin	P27824	*CANX*	144	2.5	1/3	4.5	NA	NA	NA	NA	NA
Calpain small subunit 1	P04632	*CAPNS1*	85	9	1/2	5.1	NA	NA	NA	NA	NA
Calreticulin	P27797	*CALR*	54	12	3/4	4.3	5250	5077	6465	5598	437
Casein kinase II subunit alpha 3	Q8NEV1	*CSNK2A3*	36	10	2/4	8.5	NA	NA	NA	NA	NA
Cathepsin D	P07339	*CTSD*	124	8.5	2/3	6.1	2319	3214	2599	2711	264
CD9 antigen	P21926	*CD9*	79	11	1/2	6.8	2925	3312	4269	3502	399
Cell division control protein 42 homolog	P60953	*CDC42*	238	14.1	2/12	6.2	32938	74878	43660	50492	12580
Chloride intracellular channel protein 1	O00299	*CLIC1*	113	7.5	1/2	5.1	21464	28306	21295	23689	2309
Clathrin heavy chain 1	Q00610	*CLTC*	134	6.1	7/12	5.5	NA	NA	NA	NA	NA
Cleavage and polyadenylation specificity factor subunit 6	Q16630	*CPSF6*	64	5.8	2/3	6.7	NA	NA	NA	NA	NA
Coatomer subunit delta	P48444	*ARCN1*	35	6.3	2/2	5.9	742	2651	1398	1597	560
Cofilin-1	P23528	*CFL1*	104	6.6	1/2	8.2	12392	15829	20905	16375	2472
Cofilin-2	Q9Y281	*CFL2*	60	11.4	2/3	7.7	NA	NA	NA	NA	NA
Copine-1	Q99829	*CPNE1*	89	10.2	4/11	5.5	8553	8852	6949	8118	591
Cysteine and glycine-rich protein 1	P21291	*CSRP1*	44	8.8	1/5	8.9	NA	NA	NA	NA	NA
Cysteine and histidine-rich domain-containing protein 1	Q9UHD1	*CHORDC1*	30	4.5	1/1	8.1	NA	NA	NA	NA	NA
Cytoplasmic dynein 1 heavy chain 1	Q14204	*DYNC1H1*	43	2.5	10/11	6.0	NA	NA	NA	NA	NA
Cytoskeleton-associated protein 4	Q07065	*CKAP4*	345	21.9	10/18	5.6	30415	34408	31530	32118	1189
Dedicator of cytokinesis protein 10	Q96BY6	*DOCK10*	39	2.7	7/27	6.7	30014	32499	38295	33603	2453
Dihydropyrimidinase-related protein 2	Q16555	*DPYSL2*	30	5.8	2/2	6.0	NA	NA	NA	NA	NA
DNA damage-binding protein 1	Q16531	*DDB1*	126	4.6	3/4	5.1	NA	NA	NA	NA	NA
DNA-(apurinic or apyrimidinic site) lyase	P27695	*APEX1*	83	5.3	1/2	8.3	5111	3977	6327	5138	678
Dolichyl-diphosphooligosaccharide--protein glycosyltransferase subunit 1	P04843	*RPN1*	32	7.6	4/33	6.0	8966	5549	3708	6074	1540
E3 ubiquitin/ISG15 ligase TRIM25	Q14258	*TRIM25*	36	2.9	1/1	8.4	NA	NA	NA	NA	NA
EH domain-containing protein 1	Q9H4M9	*EHD1*	31	10.5	3/5	6.4	3632	3595	4428	3885	272
EH domain-containing protein 2	Q9NZN4	*EHD2*	90	7.2	3/4	6.0	2614	1932	2495	2347	210
Elongation factor 1-alpha 1	P68104	*EEF1A1*	2090	24.5	6/196	9.1	18783	53104	48005	39964	10692
Elongation factor 1-delta	P29692	*EEF1D*	90	12.8	2/2	4.9	5966	7867	6334	6722	582
Elongation factor 2	P13639	*EEF2*	907	27.5	15/35	6.4	115949	254412	232047	200802	42915
Elongation factor Tu, mitochondrial	P49411	*TUFM*	129	15.3	4/6	7.3	79937	154101	112211	115416	21469
Endoplasmin	P14625	*HSP90B1*	325	16.2	11/21	4.8	NA	NA	NA	NA	NA
Enoyl-CoA hydratase, mitochondrial	P30084	*ECHS1*	184	13.8	3/8	8.3	10977	8799	6069	8615	1420
Eukaryotic initiation factor 4A-I	P60842	*EIF4A1*	144	14.5	4/6	5.3	7487	12602	8978	9689	1519
Eukaryotic translation initiation factor 3 subunit B	P55884	*EIF3B*	32	1.7	1/1	4.9	3901	3305	1691	2965	660
Eukaryotic translation initiation factor 3 subunit E	P60228	*EIF3E*	31	6.5	3/4	5.7	NA	NA	NA	NA	NA
Eukaryotic translation initiation factor 3 subunit H	O15372	*EIF3H*	64	5.4	1/2	6.1	688	1606	5652	2649	1525
Eukaryotic translation initiation factor 4B	P23588	*EIF4B*	40	2.8	1/1	5.6	NA	NA	NA	NA	NA
Ezrin	P15311	*EZR*	253	11.1	6/13	5.9	65257	23211	14078	34182	15759
FACT complex subunit SSRP1	Q08945	*SSRP1*	74	7.6	4/6	6.5	NA	NA	NA	NA	NA
F-actin-capping protein subunit alpha-1	P52907	*CAPZA1*	43	6.3	1/2	5.5	NA	NA	NA	NA	NA
Fascin	Q16658	*FSCN1*	24	4.9	1/1	6.8	NA	NA	NA	NA	NA
Filamin-B	O75369	*FLNB*	175	4.9	9/13	5.5	43959	39648	49052	44220	2718
Fructose-bisphosphate aldolase A	P04075	*ALDOA*	602	36.8	10/25	8.3	13903	15409	34490	21267	6626
Galectin-1	P09382	*LGALS1*	316	28.9	4/9	5.3	32143	57941	45901	45328	7453
Glucose-6-phosphate isomerase	P06744	*GPI*	96	10.2	3/7	8.4	84349	162314	141320	129328	23292
Glucosidase 2 subunit beta	P14314	*PRKCSH*	67	7.8	4/8	4.3	6655	6203	9288	7382	962
Glutathione S-transferase P	P09211	*GSTP1*	399	41.9	4/11	5.4	10280	13053	12596	11976	858
Glyceraldehyde-3-phosphate dehydrogenase	P04406	*GAPDH*	6306	54.6	15/511	8.6	393113	617970	487024	499369	65203
Glycerol kinase 2	Q14410	*GK2*	52	4.5	2/4	5.6	NA	NA	NA	NA	NA
GTP-binding nuclear protein Ran	P62826	*RAN*	69	10.6	2/4	7.0	14089	13487	13429	13668	211
HEAT repeat-containing protein 5A	Q86XA9	*HEATR5A*	32	0.4	1/209	6.1	108766	143419	102898	118361	12643
Heat shock 70 kDa protein 6	P17066	*HSPA6*	281	8.2	5/8	5.8	49191	78513	60021	62575	8560
Heat shock cognate 71 kDa protein	P11142	*HSPA8*	1032	27.6	18/41	5.4	NA	NA	NA	NA	NA
Heat shock protein beta-1	P04792	*HSPB1*	229	32.7	5/26	6.0	52530	104183	97407	84707	16207
Heat shock protein HSP 90-alpha	P07900	*HSP90AA1*	790	24.9	17/38	4.9	193399	386385	302572	294119	55870
Heat shock protein HSP 90-beta	P08238	*HSP90AB1*	664	25.8	16/38	5.0	187623	302585	285711	258640	35841
Heterogeneous nuclear ribonucleoprotein A1	P09651	*HNRNPA1*	260	18	4/13	9.2	12865	21210	15315	16463	2476
Heterogeneous nuclear ribonucleoprotein A3	P51991	*HNRNPA3*	49	8.7	2/3	9.1	NA	NA	NA	NA	NA
Heterogeneous nuclear ribonucleoprotein D0	Q14103	*HNRNPD*	88	16.9	6/10	7.6	5277	11439	5412	7376	2032
Heterogeneous nuclear ribonucleoprotein H	P31943	*HNRNPH1*	281	12.5	4/10	5.9	27089	29566	28993	28550	749
Heterogeneous nuclear ribonucleoprotein H3	P31942	*HNRNPH3*	88	4.9	1/2	6.4	2211	7723	3026	4320	1717
Heterogeneous nuclear ribonucleoprotein K	P61978	*HNRNPK*	70	11.2	4/5	5.4	5772	14163	10690	10208	2434
Heterogeneous nuclear ribonucleoprotein L	P14866	*HNRNPL*	33	3.1	1/1	8.5	NA	NA	NA	NA	NA
Heterogeneous nuclear ribonucleoprotein M	P52272	*HNRNPM*	47	7.1	4/7	8.8	8039	24508	9211	13919	5305
Heterogeneous nuclear ribonucleoprotein Q	O60506	*SYNCRIP*	82	5.3	3/7	8.7	17939	66972	51184	45365	14450
Heterogeneous nuclear ribonucleoprotein R	O43390	*HNRNPR*	143	4.1	2/7	8.2	7883	9067	10465	9138	746
Heterogeneous nuclear ribonucleoprotein U	Q00839	*HNRNPU*	127	4.7	3/6	5.8	29726	2760	38535	23674	10762
Heterogeneous nuclear ribonucleoproteins A2/B1	P22626	*HNRNPA2B1*	248	11	3/9	9.0	11555	15482	15807	14282	1366
Heterogeneous nuclear ribonucleoproteins C1/C2	P07910	*HNRNPC*	78	5.9	2/5	5.0	NA	NA	NA	NA	NA
High mobility group protein B1	P09429	*HMGB1*	45	4.2	1/2	5.6	8243	7720	8616	8193	260
High mobility group protein HMG-I/HMG-Y	P17096	*HMGA1*	51	22.4	2/3	10.3	8666	6150	12139	8985	1736
High mobility group protein HMGI-C	P52926	*HMGA2*	84	33	3/7	10.6	10373	13460	15939	13257	1610
Histone H1.0	P07305	*H1F0*	39	5.2	1/1	10.8	3673	4774	3607	4018	379
Histone H1.4	P10412	*HIST1H1E*	61	16	2/3	11.0	7118	10081	4975	7391	1480
Histone H2A type 1-B/E	P04908	*HIST1H2AB*	770	28.5	3/17	11.1	193582	270781	211052	225138	23372
Histone H2A type 1-D	P20671	*HIST1H2AD*	1000	28.5	3/21	10.9	NA	NA	NA	NA	NA
Histone H2A type 2-C	Q16777	*HIST2H2AC*	853	27.1	3/19	10.9	NA	NA	NA	NA	NA
Histone H2B type 1-B	P33778	*HIST1H2BB*	2625	34.9	4/102	10.3	NA	NA	NA	NA	NA
Histone H2B type 1-C/E/F/G/I	P62807	*HIST1H2BC*	2081	35.7	5/83	10.3	NA	NA	NA	NA	NA
Histone H2B type 1-M	Q99879	*HIST1H2BM*	2664	40.5	6/139	10.3	NA	NA	NA	NA	NA
Histone H3.1t	Q16695	*HIST3H3*	693	23.5	5/65	11.1	NA	NA	NA	NA	NA
Histone H4	P62805	*HIST1H4A*	1250	51.5	7/54	11.4	173075	247624	266794	229164	28585
Importin-7	O95373	*IPO7*	28	3.1	3/10	4.7	33398	2425	53776	29866	14929
Importin-9	Q96P70	*IPO9*	35	3.2	2/3	4.7	NA	NA	NA	NA	NA
Inosine-5′-monophosphate dehydrogenase 2	P12268	*IMPDH2*	49	7.4	4/4	6.4	5029	6715	7746	6497	792
Integrin beta-4	P16144	*ITGB4*	34	3	6/9	5.7	7980	12878	9083	9980	1483
Kelch-like protein 35	Q6PF15	*KLHL35*	139	1.2	1/80	8.1	67346	168723	114628	116899	29287
Keratin, type I cytoskeletal 18	P05783	*KRT18*	434	44.2	15/30	5.3	97751	168054	118089	127965	20887
Keratin, type II cytoskeletal 1	P04264	*KRT1*	64	4.5	3/3	8.2	7462	5503	1965	4977	1608
Keratin, type II cytoskeletal 7	P08729	*KRT7*	511	24.7	13/27	5.4	101805	143368	120907	122027	12011
Keratin, type II cytoskeletal 8	P05787	*KRT8*	621	41	24/46	5.5	179072	228296	227875	211748	16338
Lamin-B1	P20700	*LMNB1*	61	12.3	6/6	5.1	8377	11486	8587	9483	1003
Leucine--tRNA ligase, cytoplasmic	Q9P2J5	*LARS*	37	2.2	1/2	7.0	NA	NA	NA	NA	NA
l-lactate dehydrogenase A chain	P00338	*LDHA*	466	34.6	11/22	8.4	84031	140312	112944	112429	16249
l-lactate dehydrogenase B chain	P07195	*LDHB*	781	26.3	8/25	5.7	99578	162321	145095	135665	18716
Long-chain-fatty-acid--CoA ligase 4	O60488	*ACSL4*	32	4.1	2/3	8.7	1776	3441	2031	2416	518
Lysine-specific demethylase 2B	Q8NHM5	*KDM2B*	33	5.8	6/7	8.9	NA	NA	NA	NA	NA
Macrophage migration inhibitory factor	P14174	*MIF*	37	7.8	1/1	7.7	2086	18902	28020	16336	7596
Malate dehydrogenase, mitochondrial	P40926	*MDH2*	183	9.5	2/3	8.9	1723	736	1901	1453	363
Malignant T-cell-amplified sequence 1	Q9ULC4	*MCTS1*	66	9.4	1/2	9.0	11265	24343	19140	18249	3801
Matrin-3	P43243	*MATR3*	54	2.8	2/4	5.9	6219	83046	67969	52411	23503
Moesin	P26038	*MSN*	705	35.7	19/45	6.1	115884	100784	68980	95216	13823
Myosin light polypeptide 6	P60660	*MYL6*	142	29.1	3/7	4.6	NA	NA	NA	NA	NA
Myosin-9	P35579	*MYH9*	679	13.8	21/48	5.5	127807	81472	149505	119595	20064
Nascent polypeptide-associated complex subunit alpha, muscle-specific form	E9PAV3	*NACA*	168	3.6	6/8	9.6	8006	11432	6026	8488	1579
Neuroblast differentiation-associated protein AHNAK	Q09666	*AHNAK*	83	4.9	23/55	5.8	17042	32333	30396	26590	4807
Neutral alpha-glucosidase AB	Q14697	*GANAB*	44	1.4	1/1	5.7	3989	8636	9107	7244	1633
Non-POU domain-containing octamer-binding protein	Q15233	*NONO*	105	4.9	1/4	9.0	NA	NA	NA	NA	NA
Nuclear autoantigenic sperm protein	P49321	*NASP*	27	3.6	2/2	4.3	NA	NA	NA	NA	NA
Nucleolin	P19338	*NCL*	446	18.7	10/26	4.6	4183	5430	3150	4254	659
Nucleophosmin	P06748	*NPM1*	719	25.5	6/22	4.6	54142	92885	74631	73886	11190
Nucleoside diphosphate kinase A	P15531	*NME1*	135	11.2	1/4	5.8	NA	NA	NA	NA	NA
Nucleoside diphosphate kinase B	P22392	*NME2*	139	23.7	2/5	8.5	NA	NA	NA	NA	NA
Nucleosome assembly protein 1-like 1	P55209	*NAP1L1*	206	16.1	5/8	4.4	15798	27284	11672	18251	4671
Obg-like ATPase 1	Q9NTK5	*OLA1*	62	10.4	3/5	7.6	8471	6141	16052	10221	2992
Parathymosin	P20962	*PTMS*	87	20.6	2/4	4.1	NA	NA	NA	NA	NA
Peptidyl-prolyl *cis*-trans isomerase A	P62937	*PPIA*	720	62.4	10/40	7.7	245803	362998	374581	327794	41132
Peptidyl-prolyl *cis*-trans isomerase B	P23284	*PPIB*	260	22.7	4/7	9.4	30352	37989	16548	28296	6274
Peroxiredoxin-1	Q06830	*PRDX1*	219	29.1	5/10	8.3	67917	23762	24726	38801	14560
Peroxiredoxin-6	P30041	*PRDX6*	41	12.5	2/3	6.0	2287	3482	3733	3167	446
Phosphoglycerate kinase 1	P00558	*PGK1*	444	26.6	7/18	8.3	26813	36130	26255	29732	3203
Phosphoglycerate mutase 1	P18669	*PGAM1*	160	22	3/8	6.7	NA	NA	NA	NA	NA
Plasminogen activator inhibitor 1 RNA-binding protein	Q8NC51	*SERBP1*	51	5.9	2/3	8.7	5170	8322	6730	6741	910
Plasminogen activator inhibitor 2	P05120	*SERPINB2*	26	4.6	1/1	5.5	3059	5136	4166	4121	600
Plastin-3	P13797	*PLS3*	95	9.7	4/8	5.4	24969	30161	25580	26903	1638
Plectin	Q15149	*PLEC*	662	12.3	49/85	5.7	244806	307663	480157	344209	70354
Poly(rC)-binding protein 2	Q15366	*PCBP2*	69	7.4	2/3	6.3	NA	NA	NA	NA	NA
Polymerase I and transcript release factor	Q6NZI2	*CAVIN1*	225	9	3/7	5.5	11967	49975	7768	23237	13424
Polypyrimidine tract-binding protein 1	P26599	*PTBP1*	83	7.3	2/6	9.2	NA	NA	NA	NA	NA
PRA1 family protein 3	O75915	*ARL6IP5*	96	19.1	3/6	9.8	NA	NA	NA	NA	NA
Prelamin-A/C	P02545	*LMNA*	939	28.2	18/38	6.6	111642	202514	128029	147395	27963
Pre-mRNA-splicing factor CWC25 homolog	Q9NXE8	*CWC25*	31	2.4	1/2	10.2	31798	25732	41296	32942	4529
Probable ATP-dependent RNA helicase DDX17	Q92841	*DDX17*	75	6.9	5/9	8.5	2370	2019	5986	3458	1268
Probable ATP-dependent RNA helicase DDX5	P17844	*DDX5*	216	6.4	3/6	9.1	8609	85143	74034	55929	23876
Profilin-1	P07737	*PFN1*	183	59.3	7/11	8.4	23805	27917	14580	22101	3943
Prohibitin	P35232	*PHB*	35	3.7	1/1	5.6	4960	3409	2980	3783	601
Prohibitin-2	Q99623	*PHB2*	55	15.1	4/5	9.8	3673	2632	3461	3255	318
Proliferating cell nuclear antigen	P12004	*PCNA*	54	20.3	3/8	4.6	14932	17224	51964	28040	11980
Proteasome activator complex subunit 2	Q9UL46	*PSME2*	63	16.7	3/4	5.5	5726	15915	11014	10885	2942
Protein disulfide-isomerase A3	P30101	*PDIA3*	140	15.8	5/8	6.0	10505	14134	8601	11080	1623
Protein disulfide-isomerase A4	P13667	*PDIA4*	48	6.8	3/5	5.0	248455	11753	21498	93902	77328
Protein disulfide-isomerase A6	Q15084	*PDIA6*	160	16.4	4/8	5.0	18110	32619	18642	23124	4750
Protein S100-A11	P31949	*S100A11*	1067	43.8	4/29	6.6	31883	58733	83443	58020	14888
Protein S100-A6	P06703	*S100A6*	71	16.7	2/3	5.3	27347	24832	29523	27234	1355
Protein transport protein Sec61 subunit beta	P60468	*SEC61B*	35	41.7	3/3	11.6	NA	NA	NA	NA	NA
Protein-glutamine gamma-glutamyltransferase 2	P21980	*TGM2*	442	16.4	8/15	5.1	26355	43480	64930	44922	11159
Prothymosin alpha	P06454	*PTMA*	151	12.6	2/3	3.7	43185	7359	7668	19404	11891
Proto-oncogene serine/threonine-protein kinase mos	P00540	*MOS*	35	6.4	2/3	9.2	25801	73938	65324	55021	14820
Purine nucleoside phosphorylase	P00491	*PNP*	25	12.8	3/4	6.5	2740	5757	3684	4060	891
Putative nascent polypeptide-associated complex subunit alpha-like protein	Q9BZK3	*NACAP1*	31	7	1/1	4.5	6802	3279	3619	4567	1122
Putative Ras-related protein Rab-1C	Q92928	*RAB1C*	111	20.9	2/9	5.3	3926	3055	6267	4416	959
Putative UPF0633 protein MGC21881	A6NN06	*MGC21881*	32	7.4	1/8	11.9	24993	41969	21538	29500	6314
Pyruvate dehydrogenase E1 component subunit beta, mitochondrial	P11177	*PDHB*	38	4.5	1/1	6.2	4236	53498	46757	34830	15420
Pyruvate kinase PKM	P14618	*PKM*	2625	61	26/283	8.0	438545	666970	545050	550188	65991
Rab GDP dissociation inhibitor beta	P50395	*GDI2*	93	6.7	2/5	6.1	7791	26912	8837	14514	6207
Ras GTPase-activating-like protein IQGAP1	P46940	*IQGAP1*	62	5.7	7/12	6.1	1399	4811	7389	4533	1735
Ras-related protein Rab-1B	Q9H0U4	*RAB1B*	115	8.5	1/4	5.6	NA	NA	NA	NA	NA
Ras-related protein Rab-5C	P51148	*RAB5C*	43	19	2/2	8.6	6423	5976	3432	5277	932
Receptor of activated protein C kinase 1	P63244	*RACK1*	41	7.6	2/4	7.6	2447	4432	4549	3810	682
Rho GDP-dissociation inhibitor 1	P52565	*ARHGDIA*	125	7.4	1/2	5.0	3282	5236	4556	4358	573
Rho GDP-dissociation inhibitor 2	P52566	*ARHGDIB*	46	12.9	1/1	5.1	NA	NA	NA	NA	NA
Rho GTPase-activating protein 26	Q9UNA1	*ARHGAP26*	40	2.9	2/8	6.2	NA	NA	NA	NA	NA
Ribonuclease inhibitor	P13489	*RNH1*	93	18.2	5/7	4.7	33810	78512	50934	54419	13021
RNA polymerase II-associated protein 1	Q9BWH6	*RPAP1*	29	2.3	3/4	6.0	5359	11227	3656	6747	2293
RNA-binding motif protein, X chromosome	P38159	*RBMX*	41	13.8	4/5	10.1	9419	5591	3728	6246	1675
RNA-binding protein FUS	P35637	*FUS*	262	7.8	2/4	9.4	NA	NA	NA	NA	NA
Serpin H1	P50454	*SERPINH1*	126	8.9	2/6	8.8	21058	54281	40568	38635	9639
SH3 domain-binding glutamic acid-rich-like protein 3	Q9H299	*SH3BGRL3*	39	28	2/3	4.8	NA	NA	NA	NA	NA
Signal recognition particle 14 kDa protein	P37108	*SRP14*	171	10.3	1/2	10.1	NA	NA	NA	NA	NA
Small nuclear ribonucleoprotein Sm D1	P62314	*SNRPD1*	34	16.8	1/4	11.6	4099	40358	18953	21136	10524
Sorting nexin-6	Q9UNH7	*SNX6*	35	5.2	1/1	5.8	NA	NA	NA	NA	NA
Splicing factor 3B subunit 1	O75533	*SF3B1*	31	4.1	4/9	6.7	NA	NA	NA	NA	NA
Splicing factor, proline- and glutamine-rich	P23246	*SFPQ*	145	7.2	3/6	9.5	2648	2244	2899	2597	191
Stathmin	P16949	*STMN1*	36	6	1/1	5.8	5573	3734	6320	5209	768
Stress-70 protein, mitochondrial	P38646	*HSPA9*	108	14.9	8/12	5.9	39027	56023	66009	53686	7876
Stress-induced-phosphoprotein 1	P31948	*STIP1*	34	5.5	3/3	6.4	2244	5934	2006	3395	1272
Sulfotransferase 1A3	P0DMM9	*SULT1A3*	69	11.2	2/4	5.7	NA	NA	NA	NA	NA
Surfeit locus protein 4	O15260	*SURF4*	87	6.7	1/4	7.6	NA	NA	NA	NA	NA
T-complex protein 1 subunit beta	P78371	*CCT2*	33	18.9	5/5	6.0	NA	NA	NA	NA	NA
T-complex protein 1 subunit delta	P50991	*CCT4*	110	10.6	5/10	8.0	4676	5444	2479	4200	888
T-complex protein 1 subunit eta	Q99832	*CCT7*	207	15.3	4/11	7.6	NA	NA	NA	NA	NA
T-complex protein 1 subunit gamma	P49368	*CCT3*	48	15	4/5	6.1	NA	NA	NA	NA	NA
T-complex protein 1 subunit theta	P50990	*CCT8*	108	8.8	3/6	5.4	45589	106359	68974	73641	17697
T-complex protein 1 subunit zeta	P40227	*CCT6A*	132	14.5	5/8	6.2	28802	41737	31766	34102	3912
Thioredoxin domain-containing protein 5	Q8NBS9	*TXNDC5*	210	13	4/8	5.6	3429	38778	6213	16140	11348
Thioredoxin reductase 1, cytoplasmic	Q16881	*TXNRD1*	99	4.8	2/3	7.2	NA	NA	NA	NA	NA
Transaldolase	P37837	*TALDO1*	31	5	2/3	6.4	16133	8649	10676	11819	2235
Transcription elongation factor A protein-like 3	Q969E4	*TCEAL3*	37	20	2/6	4.9	NA	NA	NA	NA	NA
Transcription factor BTF3	P20290	*BTF3*	25	10.7	1/1	9.4	NA	NA	NA	NA	NA
Transcription intermediary factor 1-beta	Q13263	*TRIM28*	54	5.4	3/5	5.5	125189	119123	87448	110587	11701
Transgelin-2	P37802	*TAGLN2*	752	49.7	8/26	8.4	92336	122765	74851	96651	13999
Transitional endoplasmic reticulum ATPase	P55072	*VCP*	221	7.1	4/20	5.1	33971	43452	35614	37679	2925
Transketolase	P29401	*TKT*	604	15.4	8/18	7.6	33590	51876	52238	45901	6157
Triosephosphate isomerase	P60174	*TPI1*	781	57	10/26	5.7	176607	191403	200103	189371	6858
Tropomodulin-3	Q9NYL9	*TMOD3*	39	9.7	2/2	5.1	3993	6618	942	3851	1640
Tropomyosin alpha-4 chain	P67936	*TPM4*	140	28.6	7/13	4.7	NA	NA	NA	NA	NA
Tubulin alpha-1B chain	P68363	*TUBA1B*	750	31.9	9/31	4.9	154337	286347	229566	223417	38232
Tubulin beta chain	P07437	*TUBB*	916	25.7	9/33	4.8	103573	191973	138370	144638	25711
Tubulin beta-4A chain	P04350	*TUBB4A*	535	23	8/22	4.8	137521	76265	134509	116098	19935
Tubulin beta-4B chain	P68371	*TUBB4B*	601	25.2	10/24	4.8	NA	NA	NA	NA	NA
Tubulin beta-6 chain	Q9BUF5	*TUBB6*	348	23.3	8/17	4.8	35795	43771	55976	45181	5868
Ubiquitin carboxyl-terminal hydrolase isozyme L1	P09936	*UCHL1*	62	36.8	5/5	5.3	11726	30518	24641	22295	5550
Ubiquitin-40S ribosomal protein S27a	P62979	*RPS27A*	340	31.4	4/12	9.7	NA	NA	NA	NA	NA
Ubiquitin-like modifier-activating enzyme 1	P22314	*UBA1*	280	5.9	4/9	5.5	25291	32044	34487	30607	2750
UMP-CMP kinase	P30085	*CMPK1*	34	17.9	2/3	5.4	NA	NA	NA	NA	NA
UPF0183 protein C16orf70	Q9BSU1	*C16orf70*	32	6.6	2/5	7.6	33064	54344	36724	41377	6569
UPF0258 protein KIAA1024	Q9UPX6	*KIAA1024*	36	4	3/6	7.0	7991	12747	2359	7699	3003
Vesicle-trafficking protein SEC22b	O75396	*SEC22B*	46	10.2	2/3	6.4	NA	NA	NA	NA	NA
Vimentin	P08670	*VIM*	3159	59.4	30/296	5.1	625554	1135631	907520	889568	147520
Vinculin	P18206	*VCL*	71	7	6/10	5.5	3928	3134	4831	3964	490
Voltage-dependent anion-selective channel protein 1	P21796	*VDAC1*	53	17	3/5	8.6	4957	1860	7036	4618	1504
X-ray repair cross-complementing protein 5	P13010	*XRCC5*	78	5.2	2/4	5.6	9578	18903	10299	12927	2995
X-ray repair cross-complementing protein 6	P12956	*XRCC6*	115	11.3	5/9	6.2	3537	2660	5836	4011	947
Zyxin	Q15942	*ZYX*	80	4.5	2/3	6.2	3809	3137	4853	3933	499

%Cov = %Sequence coverage = (number of the matched residues/total number of residues in the entire sequence) x 100%.

NA = not applicable (protein was identified in the sample but its MS/MS spectra did not meet predefined criteria for high-confident intensity analysis).

**Table 2:** All proteins identified in caffeine-exposed EA.hy926 endothelial cells.

Protein name	Swiss-Prot ID	Gene symbol	MS/MS identification score	% Cov	No. of distinct/total matched peptides	p*I*	Intensity (x10^3^ arbitrary unit)				
							N1	N2	N3	Mean	SEM
10 kDa heat shock protein, mitochondrial	P61604	*HSPE1*	44	6.9	1/2	8.9	4330	3885	4178	4131	131
14-3-3 protein beta/alpha	P31946	*YWHAB*	142	12.2	2/5	4.8	20109	24727	21213	22016	1392
14-3-3 protein epsilon	P62258	*YWHAE*	142	13.7	2/5	4.6	5748	5258	5227	5411	169
14-3-3 protein theta	P27348	*YWHAQ*	112	8.2	2/4	4.7	29509	33111	31173	31264	1041
14-3-3 protein zeta/delta	P63104	*YWHAZ*	149	30.6	6/14	4.7	18925	23312	15327	19188	2309
26S protease regulatory subunit 6B	P43686	*PSMC4*	32	6.9	3/3	5.1	43621	52820	44514	46985	2929
26S protease regulatory subunit 7	P35998	*PSMC2*	43	9	3/4	5.7	13867	19513	13205	15528	2002
26S proteasome non-ATPase regulatory subunit 12	O00232	*PSMD12*	37	9.6	3/7	7.5	23918	34643	33745	30769	3435
26S proteasome non-ATPase regulatory subunit 14	O00487	*PSMD14*	44	21.3	2/3	6.1	NA	NA	NA	NA	NA
3-ketoacyl-CoA thiolase, mitochondrial	P42765	*ACAA2*	24	6.5	1/1	8.3	NA	NA	NA	NA	NA
40S ribosomal protein S11	P62280	*RPS11*	36	13.3	2/2	10.3	4709	6986	5917	5871	658
40S ribosomal protein S12	P25398	*RPS12*	109	28	3/6	6.8	18285	10178	13333	13932	2359
40S ribosomal protein S13	P62277	*RPS13*	129	18.5	3/7	10.5	655	5388	5511	3851	1599
40S ribosomal protein S14	P62263	*RPS14*	129	21.2	3/5	10.1	30601	36697	31621	32973	1885
40S ribosomal protein S15	P62841	*RPS15*	74	21.4	2/3	10.4	7814	19633	5966	11138	4281
40S ribosomal protein S16	P62249	*RPS16*	131	14.4	2/4	10.2	23146	41588	25518	30084	5793
40S ribosomal protein S18	P62269	*RPS18*	67	18.4	2/3	11.0	5993	6056	7327	6458	435
40S ribosomal protein S19	P39019	*RPS19*	59	12.4	2/4	10.3	9850	13001	10722	11191	939
40S ribosomal protein S2	P15880	*RPS2*	128	17.1	3/5	10.3	24603	15691	28391	22895	3764
40S ribosomal protein S20	P60866	*RPS20*	85	10.1	1/4	10.0	7278	7636	8997	7971	524
40S ribosomal protein S25	P62851	*RPS25*	78	8	1/3	10.1	2534	3395	7205	4378	1435
40S ribosomal protein S3	P23396	*RPS3*	69	10.7	3/5	9.7	4920	5693	5855	5489	288
40S ribosomal protein S30	P62861	*FAU*	46	16.9	1/2	12.2	6266	4765	8417	6483	1060
40S ribosomal protein S3a	P61247	*RPS3A*	47	14.8	3/5	9.8	14393	12075	7992	11487	1871
40S ribosomal protein S4, X isoform	P62701	*RPS4X*	49	25.1	5/7	10.2	9693	8466	7339	8500	680
40S ribosomal protein S5	P46782	*RPS5*	746	8.8	2/14	9.7	20193	83956	64212	56120	18846
40S ribosomal protein S6	P62753	*RPS6*	54	12.9	3/4	10.9	3336	7375	9951	6887	1925
40S ribosomal protein S7	P62081	*RPS7*	97	23.7	4/5	10.1	6560	9532	10157	8749	1110
40S ribosomal protein S8	P62241	*RPS8*	38	5.3	1/1	10.3	6473	10745	5264	7494	1663
40S ribosomal protein SA	P08865	*RPSA*	110	14.9	2/3	4.8	2129	3513	5312	3651	921
60 kDa heat shock protein, mitochondrial	P10809	*HSPD1*	1040	18.3	7/32	5.7	19242	34047	64047	39112	13180
60S acidic ribosomal protein P0-like	Q8NHW5	*RPLP0P6*	72	18	4/6	5.4	NA	NA	NA	NA	NA
60S acidic ribosomal protein P1	P05386	*RPLP1*	33	14	1/1	4.3	43660	60149	35480	46430	7255
60S acidic ribosomal protein P2	P05387	*RPLP2*	55	39.1	2/5	4.4	12111	19706	23420	18413	3328
60S ribosomal protein L10	P27635	*RPL10*	108	12.1	2/3	10.1	6324	13597	77703	32541	22678
60S ribosomal protein L12	P30050	*RPL12*	114	14.5	2/3	9.5	6275	7716	6677	6889	429
60S ribosomal protein L17	P18621	*RPL17*	36	14.1	3/5	10.2	8719	6548	9066	8111	788
60S ribosomal protein L18	Q07020	*RPL18*	43	14.4	2/2	11.7	3933	6316	8707	6319	1378
60S ribosomal protein L19	P84098	*RPL19*	70	13.3	2/2	11.5	76851	24607	75253	58904	17155
60S ribosomal protein L23	P62829	*RPL23*	128	14.3	1/3	10.5	5526	7317	10014	7619	1304
60S ribosomal protein L23a	P62750	*RPL23A*	42	13.5	2/2	10.4	8990	17173	14570	13578	2414
60S ribosomal protein L27	P61353	*RPL27*	39	13.2	2/3	10.6	8472	3960	3332	5255	1619
60S ribosomal protein L29	P47914	*RPL29*	49	9.4	1/2	11.7	7850	4868	6524	6414	863
60S ribosomal protein L30	P62888	*RPL30*	93	20	2/3	9.7	10204	31234	11231	17556	6845
60S ribosomal protein L36	Q9Y3U8	*RPL36*	70	19	2/2	11.6	20307	26460	24430	23732	1810
60S ribosomal protein L38	P63173	*RPL38*	107	35.7	2/9	10.1	NA	NA	NA	NA	NA
60S ribosomal protein L4	P36578	*RPL4*	35	6.3	2/3	11.1	8221	6733	9680	8212	851
60S ribosomal protein L6	Q02878	*RPL6*	39	6.3	2/3	10.6	19103	4896	5498	9832	4639
60S ribosomal protein L7	P18124	*RPL7*	84	14.9	2/4	10.7	NA	NA	NA	NA	NA
60S ribosomal protein L7a	P62424	*RPL7A*	27	14.7	3/3	10.6	2578	2984	2804	2788	118
78 kDa glucose-regulated protein	P11021	*HSPA5*	824	32.3	19/39	5.1	NA	NA	NA	NA	NA
Acidic leucine-rich nuclear phosphoprotein 32 family member A	P39687	*ANP32A*	102	8.4	1/4	4.0	NA	NA	NA	NA	NA
Actin, aortic smooth muscle	P62736	*ACTA2*	2034	26.8	12/198	5.2	NA	NA	NA	NA	NA
Activator of 90 kDa heat shock protein ATPase homolog 1	O95433	*AHSA1*	27	6.5	1/3	5.4	NA	NA	NA	NA	NA
Adenylyl cyclase-associated protein 1	Q01518	*CAP1*	39	11.2	3/8	8.2	NA	NA	NA	NA	NA
ADP/ATP translocase 2	P05141	*SLC25A5*	68	15.8	5/16	9.7	40781	41979	38952	40571	880
ADP-ribosylation factor 1	P84077	*ARF1*	34	16	2/3	6.3	NA	NA	NA	NA	NA
Alpha-actinin-1	P12814	*ACTN1*	219	9.4	6/9	5.3	NA	NA	NA	NA	NA
Alpha-actinin-4	O43707	*ACTN4*	345	17.2	12/15	5.3	259893	117977	96297	158056	51302
Alpha-enolase	P06733	*ENO1*	1797	46.5	14/215	7.0	160580	195706	188976	181754	10764
Ankyrin repeat domain-containing protein SOWAHA	Q2M3V2	*SOWAHA*	30	4.4	2/97	10.2	83308	87565	65250	78708	6840
Annexin A1	P04083	*ANXA1*	887	39	10/28	6.6	90580	130768	121496	114281	12149
Annexin A2	P07355	*ANXA2*	2306	51.9	16/190	7.6	NA	NA	NA	NA	NA
Annexin A5	P08758	*ANXA5*	1474	35.9	11/44	4.9	248633	339406	258133	282057	28805
ATP synthase subunit alpha, mitochondrial	P25705	*ATP5F1A*	296	6.7	2/7	9.2	85448	65941	111158	87515	13094
ATP synthase subunit beta, mitochondrial	P06576	*ATP5F1B*	540	27	10/39	5.3	26094	26613	22905	25204	1159
ATP-dependent RNA helicase DDX1	Q92499	*DDX1*	99	5.9	2/4	6.8	NA	NA	NA	NA	NA
ATP-dependent RNA helicase DDX3X	O00571	*DDX3X*	100	4.8	2/4	6.7	NA	NA	NA	NA	NA
Barrier-to-autointegration factor	O75531	*BANF1*	54	27	1/2	5.8	NA	NA	NA	NA	NA
Basic leucine zipper and W2 domain-containing protein 1	Q7L1Q6	*BZW1*	164	7.4	2/5	5.8	NA	NA	NA	NA	NA
Beta-actin-like protein 2	Q562R1	*ACTBL2*	1403	17.8	7/275	5.4	271784	100047	361485	244439	76699
Calnexin	P27824	*CANX*	103	2.5	1/4	4.5	NA	NA	NA	NA	NA
Calpain small subunit 1	P04632	*CAPNS1*	46	9	1/2	5.1	NA	NA	NA	NA	NA
Calpain-2 catalytic subunit	P17655	*CAPN2*	30	2.1	1/1	4.9	7615	12113	9347	9692	1310
Calreticulin	P27797	*CALR*	43	12	3/5	4.3	4362	4714	4586	4554	103
Casein kinase II subunit alpha	P68400	*CSNK2A1*	33	4.6	1/2	7.3	NA	NA	NA	NA	NA
Cathepsin D	P07339	*CTSD*	156	8.5	2/6	6.1	19350	26988	15663	20667	3335
Caveolin-1	Q03135	*CAV1*	114	25.3	3/5	5.7	16891	9077	14234	13401	2294
CD59 glycoprotein	P13987	*CD59*	45	9.4	1/3	6.0	6779	15002	11515	11099	2383
Cell division control protein 42 homolog	P60953	*CDC42*	168	19.9	3/10	6.2	22862	38185	24789	28612	4819
Centriolin	Q7Z7A1	*CNTRL*	29	4.1	8/14	5.4	NA	NA	NA	NA	NA
Chloride intracellular channel protein 1	O00299	*CLIC1*	126	14.5	2/4	5.1	35230	32390	31734	33118	1073
Clathrin heavy chain 1	Q00610	*CLTC*	111	4.7	5/8	5.5	NA	NA	NA	NA	NA
Cleavage and polyadenylation specificity factor subunit 6	Q16630	*CPSF6*	73	8.7	3/3	6.7	NA	NA	NA	NA	NA
Coatomer subunit gamma-1	Q9Y678	*COPG1*	40	2.5	2/2	5.3	NA	NA	NA	NA	NA
Cofilin-1	P23528	*RPS3*	89	15.1	2/3	8.2	17057	11077	11607	13247	1911
Complement component C8 alpha chain	P07357	*C8A*	41	2.9	1/2	6.1	8969	14398	11483	11617	1569
Copine-1	Q99829	*CPNE1*	112	10.2	4/26	5.5	5980	6341	15281	9201	3042
Core histone macro-H2A.1	O75367	*H2AFY*	28	8.1	2/2	9.8	NA	NA	NA	NA	NA
Cytoskeleton-associated protein 4	Q07065	*CKAP4*	297	20.1	8/14	5.6	38642	64756	67614	57004	9218
Dehydrogenase/reductase SDR family member 12	A0PJE2	*DHRS12*	37	2.5	1/1	6.8	NA	NA	NA	NA	NA
Dihydropyrimidinase-related protein 1	Q14194	*CRMP1*	36	10.5	4/5	6.6	36379	2686	34659	24575	10956
DNA damage-binding protein 1	Q16531	*DDB1*	103	4.6	3/5	5.1	NA	NA	NA	NA	NA
DNA replication licensing factor MCM3	P25205	*MCM3*	46	8.9	5/8	5.5	NA	NA	NA	NA	NA
DNA replication licensing factor MCM4	P33991	*MCM4*	30	5.2	3/5	6.3	NA	NA	NA	NA	NA
DNA-(apurinic or apyrimidinic site) lyase	P27695	*APEX1*	39	17.6	3/4	8.3	2478	5382	6307	4722	1153
DNA-dependent protein kinase catalytic subunit	P78527	*PRKDC*	30	3.4	11/25	6.8	NA	NA	NA	NA	NA
Dolichyl-diphosphooligosaccharide--protein glycosyltransferase subunit 1	P04843	*RPN1*	43	4.4	2/3	6.0	39756	47552	45760	44356	2357
Dolichyl-diphosphooligosaccharide--protein glycosyltransferase subunit 2	P04844	*RPN2*	37	1.9	1/1	5.4	3259	1785	4937	3327	910
E3 ubiquitin/ISG15 ligase TRIM25	Q14258	*TRIM25*	33	2.9	1/1	8.4	NA	NA	NA	NA	NA
EH domain-containing protein 1	Q9H4M9	*EHD1*	28	4.3	1/1	6.4	2850	2771	2456	2693	120
Elongation factor 1-alpha 1	P68104	*EEF1A1*	1816	27.1	7/200	9.1	81440	79986	83219	81548	935
Elongation factor 1-delta	P29692	*EEF1D*	60	6.8	2/3	4.9	4253	3855	3494	3868	219
Elongation factor 1-gamma	P26641	*EEF1G*	36	11.9	4/8	6.3	20221	9537	15958	15239	3105
Elongation factor 2	P13639	*EEF2*	766	26.3	14/41	6.4	154712	236101	219802	203538	24862
Elongation factor Tu, mitochondrial	P49411	*TUFM*	144	19	5/7	7.3	22502	131221	92707	82144	31826
Endoplasmin	P14625	*HSP90B1*	330	10.2	7/19	4.8	NA	NA	NA	NA	NA
Enoyl-CoA hydratase, mitochondrial	P30084	*ECHS1*	114	5.9	1/5	8.3	3923	4257	6914	5032	946
Eukaryotic initiation factor 4A-I	P60842	*EIF4A1*	194	17.7	5/9	5.3	4303	13016	11882	9734	2735
Eukaryotic translation initiation factor 3 subunit E	P60228	*EIF3E*	39	4.3	1/1	5.7	NA	NA	NA	NA	NA
Eukaryotic translation initiation factor 3 subunit F	O00303	*EIF3F*	89	5.3	1/5	5.2	5340	3388	6309	5012	859
Eukaryotic translation initiation factor 4B	P23588	*EIF4B*	43	2.8	1/2	5.6	NA	NA	NA	NA	NA
Ezrin	P15311	*EZR*	292	9.6	6/18	5.9	19984	39956	39871	33270	6643
FACT complex subunit SSRP1	Q08945	*SSRP1*	29	8.9	4/5	6.5	NA	NA	NA	NA	NA
F-actin-capping protein subunit alpha-1	P52907	*CAPZA1*	42	6.3	1/2	5.5	NA	NA	NA	NA	NA
F-actin-capping protein subunit beta	P47756	*CAPZB*	80	9	1/1	5.4	NA	NA	NA	NA	NA
Far upstream element-binding protein 2	Q92945	*KHSRP*	30	1.4	1/1	6.9	2971	3055	4257	3428	415
Fascin	Q16658	*FSCN1*	25	4.9	1/1	6.8	NA	NA	NA	NA	NA
Filamin-A	P21333	*FLNA*	499	11.9	19/28	5.7	NA	NA	NA	NA	NA
Filamin-B	O75369	*FLNB*	92	2.1	4/6	5.5	41775	44092	39521	41796	1320
Fructose-bisphosphate aldolase A	P04075	*ALDOA*	518	34.9	9/24	8.3	34570	39782	36191	36848	1540
Galectin-1	P09382	*LGALS1*	380	42.2	5/16	5.3	51517	52333	58343	54064	2152
Gamma-interferon-inducible protein 16	Q16666	*IFI16*	33	7.4	4/5	9.3	NA	NA	NA	NA	NA
Glucose-6-phosphate isomerase	P06744	*GPI*	77	10.2	3/5	8.4	45245	67097	59458	57267	6403
Glucosidase 2 subunit beta	P14314	*PRKCSH*	58	7.8	4/5	4.3	16495	19203	14906	16868	1254
Glutamate dehydrogenase 1, mitochondrial	P00367	*GLUD1*	74	8.2	3/5	7.7	NA	NA	NA	NA	NA
Glutathione S-transferase P	P09211	*GSTP1*	303	41.9	4/8	5.4	10233	11386	11396	11005	386
Glyceraldehyde-3-phosphate dehydrogenase	P04406	*GAPDH*	6709	54.9	16/338	8.6	502087	483920	504326	496778	6461
Glycerol kinase 2	Q14410	*GK2*	40	1.6	1/5	5.6	NA	NA	NA	NA	NA
GTP-binding nuclear protein Ran	P62826	*RAN*	82	15.7	3/5	7.0	49761	47473	54665	50633	2121
HEAT repeat-containing protein 5A	Q86XA9	*HEATR5A*	26	1.1	2/134	6.1	83125	92266	68546	81312	6907
Heat shock 70 kDa protein 1A	P0DMV8	*HSPA1A*	224	10.3	5/9	5.5	66770	61785	54021	60859	3709
Heat shock 70 kDa protein 6	P17066	*HSPA6*	308	9.5	6/12	5.8	64974	60904	59196	61691	1714
Heat shock cognate 71 kDa protein	P11142	*HSPA8*	908	31.6	18/37	5.4	NA	NA	NA	NA	NA
Heat shock protein beta-1	P04792	*HSPB1*	241	37.6	6/12	6.0	43768	32291	27387	34482	4854
Heat shock protein HSP 90-alpha	P07900	*HSP90AA1*	722	28.1	15/40	4.9	243625	290440	265009	266358	13531
Heat shock protein HSP 90-beta	P08238	*HSP90AB1*	774	31.4	17/38	5.0	183622	213245	204446	200438	8783
Heterogeneous nuclear ribonucleoprotein D0	Q14103	*HNRNPD*	66	14.4	4/6	7.6	3453	6167	6846	5489	1037
Heterogeneous nuclear ribonucleoprotein H	P31943	*HNRNPH1*	254	12.5	4/9	5.9	13440	18911	24674	19008	3243
Heterogeneous nuclear ribonucleoprotein H3	P31942	*HNRNPH3*	99	4.9	1/3	6.4	4010	3124	4367	3834	370
Heterogeneous nuclear ribonucleoprotein K	P61978	*HNRNPK*	138	11.2	4/7	5.4	18919	15369	21312	18534	1726
Heterogeneous nuclear ribonucleoprotein L	P14866	*HNRNPL*	34	3.1	1/1	8.5	NA	NA	NA	NA	NA
Heterogeneous nuclear ribonucleoprotein M	P52272	*HNRNPM*	58	14.8	7/9	8.8	4171	19389	10860	11473	4404
Heterogeneous nuclear ribonucleoprotein Q	O60506	*SYNCRIP*	64	7.1	3/6	8.7	43372	33645	26888	34635	4784
Heterogeneous nuclear ribonucleoprotein R	O43390	*HNRNPR*	91	4.1	2/3	8.2	5230	7021	12551	8268	2203
Heterogeneous nuclear ribonucleoprotein U	Q00839	*HNRNPU*	81	9.8	6/9	5.8	7785	7811	8959	8185	387
Heterogeneous nuclear ribonucleoproteins A2/B1	P22626	*HNRNPA2B1*	264	14.7	4/8	9.0	9234	7503	13746	10161	1861
Heterogeneous nuclear ribonucleoproteins C1/C2	P07910	*HNRNPC*	74	5.9	2/3	5.0	NA	NA	NA	NA	NA
High mobility group protein HMGI-C	P52926	*HMGA2*	145	33	3/9	10.6	6710	7154	5082	6315	630
Histone H1.4	P10412	*HIST1H1E*	187	21.5	4/9	11.0	29860	87832	90815	69502	19840
Histone H1.5	P16401	*HIST1H1B*	38	10.6	2/2	10.9	13058	22109	11606	15591	3286
Histone H2A type 1-B/E	P04908	*HIST1H2AB*	1824	28.5	3/214	11.1	251738	258348	301719	270602	15675
Histone H2A type 1-D	P20671	*HIST1H2AD*	1914	28.5	3/216	10.9	NA	NA	NA	NA	NA
Histone H2B type 1-B	P33778	*HIST1H2BB*	7978	34.9	4/395	10.3	NA	NA	NA	NA	NA
Histone H2B type 1-C/E/F/G/I	P62807	*HIST1H2BC*	7971	35.7	5/400	10.3	NA	NA	NA	NA	NA
Histone H2B type 1-M	Q99879	*HIST1H2BM*	2783	52.4	8/95	10.3	NA	NA	NA	NA	NA
Histone H3.1t	Q16695	*HIST3H3*	218	19.1	3/16	11.1	NA	NA	NA	NA	NA
Histone H4	P62805	*HIST1H4A*	1780	51.5	7/88	11.4	350255	303112	359011	337459	17359
Hsc70-interacting protein	P50502	*ST13*	30	5.1	2/2	5.2	3011	2942	1149	2367	609
Importin subunit beta-1	Q14974	*KPNB1*	30	3.4	2/3	4.7	32953	41673	1683	25436	12140
Importin-9	Q96P70	*IPO9*	68	3.5	2/6	4.7	NA	NA	NA	NA	NA
Integrin beta-4	P16144	*ITGB4*	44	3.7	6/8	5.7	4247	13505	11076	9609	2771
Interleukin enhancer-binding factor 2	Q12905	*ILF2*	27	8.7	2/3	5.2	1288	7257	7991	5512	2123
Interleukin enhancer-binding factor 3	Q12906	*ILF3*	64	7.3	5/5	8.9	2673	3160	3173	3002	164
Kelch-like protein 35	Q6PF15	*KLHL35*	76	3.9	2/54	8.1	90822	94586	123950	103119	10472
Keratin, type II cytoskeletal 7	P08729	*KRT7*	456	20.3	9/25	5.4	110541	140404	132067	127671	8896
Keratin, type II cytoskeletal 8	P05787	*KRT8*	643	33.1	16/34	5.5	168002	180131	185018	177717	5058
l-lactate dehydrogenase A chain	P00338	*LDHA*	384	25.9	8/20	8.4	113122	114854	122830	116936	2989
l-lactate dehydrogenase B chain	P07195	*LDHB*	793	35.3	9/22	5.7	151327	164173	149031	154844	4712
Lysine-specific demethylase 2B	Q8NHM5	*KDM2B*	31	5.2	5/6	8.9	NA	NA	NA	NA	NA
Moesin	P26038	*MSN*	765	31.2	17/46	6.1	75143	104685	121996	100608	13678
Myosin light polypeptide 6	P60660	*MYL6*	145	19.2	2/5	4.6	NA	NA	NA	NA	NA
Myosin-9	P35579	*MYH9*	606	15.1	23/44	5.5	136722	154816	75160	122233	24109
Myristoylated alanine-rich C-kinase substrate	P29966	*MARCKS*	100	11.4	2/3	4.5	NA	NA	NA	NA	NA
Nascent polypeptide-associated complex subunit alpha, muscle-specific form	E9PAV3	*NACA*	64	2.5	3/3	9.6	4236	6604	4831	5224	711
Neurobeachin-like protein 2	Q6ZNJ1	*NBEAL2*	26	0.9	3/5	6.0	NA	NA	NA	NA	NA
Neuroblast differentiation-associated protein AHNAK	Q09666	*AHNAK*	106	3.9	14/28	5.8	31540	28337	24712	28197	1972
Nuclear autoantigenic sperm protein	P49321	*NASP*	74	2.9	1/2	4.3	NA	NA	NA	NA	NA
Nucleolin	P19338	*NCL*	340	12.4	5/14	4.6	18999	29968	26172	25046	3216
Nucleophosmin	P06748	*NPM1*	560	25.5	6/22	4.6	70290	87003	71940	76411	5317
Nucleoside diphosphate kinase A	P15531	*NME1*	256	28.9	3/9	5.8	NA	NA	NA	NA	NA
Nucleoside diphosphate kinase B	P22392	*NME2*	110	23.7	2/5	8.5	NA	NA	NA	NA	NA
Nucleosome assembly protein 1-like 1	P55209	*NAP1L1*	244	11.8	3/9	4.4	14361	22476	19359	18732	2363
Obg-like ATPase 1	Q9NTK5	*OLA1*	65	7.6	2/3	7.6	11814	7151	3737	7567	2341
Parathymosin	P20962	*PTMS*	42	11.8	1/1	4.1	NA	NA	NA	NA	NA
Peptidyl-prolyl *cis*-trans isomerase A	P62937	*PPIA*	855	58.2	8/44	7.7	231705	300387	236591	256227	22125
Peptidyl-prolyl *cis*-trans isomerase B	P23284	*PPIB*	175	28.2	5/8	9.4	12703	18919	14342	15322	1860
Peroxiredoxin-1	Q06830	*PRDX1*	206	48.7	8/15	8.3	27647	30113	35543	31101	2332
Peroxiredoxin-6	P30041	*PRDX6*	54	22.3	3/6	6.0	1613	1696	2904	2071	417
Phenylalanine--tRNA ligase alpha subunit	Q9Y285	*FARSA*	31	7.5	3/4	7.3	11640	18021	14123	14595	1857
Phosphoglycerate kinase 1	P00558	*PGK1*	468	30.9	9/19	8.3	35780	29231	26549	30520	2741
Phosphoglycerate mutase 1	P18669	*PGAM1*	154	22	3/7	6.7	NA	NA	NA	NA	NA
Plasminogen activator inhibitor 2	P05120	*SERPINB2*	40	6.7	2/3	5.5	10671	46264	15720	24218	11119
Plectin	Q15149	*PLEC*	629	10.1	40/76	5.7	129537	94526	112414	112159	10108
Poly(rC)-binding protein 2	Q15366	*PCBP2*	143	10.4	3/5	6.3	NA	NA	NA	NA	NA
Polymerase I and transcript release factor	Q6NZI2	*CAVIN1*	203	7.7	2/4	5.5	18569	26062	26052	23561	2496
Polypyrimidine tract-binding protein 1	P26599	*PTBP1*	77	8.1	2/3	9.2	NA	NA	NA	NA	NA
PRA1 family protein 3	O75915	*ARL6IP5*	75	10.1	1/2	9.8	NA	NA	NA	NA	NA
Prelamin-A/C	P02545	*LMNA*	801	30.4	18/42	6.6	178301	195963	179349	184538	5721
Probable ATP-dependent RNA helicase DDX17	Q92841	*DDX17*	70	7.8	6/11	8.5	10037	3635	7211	6961	1852
Probable ATP-dependent RNA helicase DDX5	P17844	*DDX5*	160	9.1	4/6	9.1	60887	75245	68979	68370	4156
Profilin-1	P07737	*PFN1*	173	29.3	4/11	8.4	25682	31776	35538	30999	2871
Prohibitin	P35232	*PHB*	60	3.7	1/3	5.6	3563	3755	3652	3657	56
Prohibitin-2	Q99623	*PHB2*	84	6	2/3	9.8	3060	11607	4513	6393	2640
Proliferating cell nuclear antigen	P12004	*PCNA*	89	18.8	3/5	4.6	10521	15864	15787	14057	1768
Proliferation-associated protein 2G4	Q9UQ80	*PA2G4*	60	13.5	4/5	6.1	15606	4851	5579	8679	3470
Proteasome activator complex subunit 2	Q9UL46	*PSME2*	84	5.4	1/3	5.5	1510	6784	13337	7211	3421
Protein disulfide-isomerase A3	P30101	*PDIA3*	198	13.5	5/9	6.0	14479	24142	14370	17664	3239
Protein disulfide-isomerase A4	P13667	*PDIA4*	60	1.9	1/2	5.0	1248	3013	3897	2719	779
Protein disulfide-isomerase A6	Q15084	*PDIA6*	103	16.4	4/7	5.0	15804	36195	31234	27744	6139
Protein MCM10 homolog	Q7L590	*MCM10*	20	2.6	2/16	9.0	NA	NA	NA	NA	NA
Protein S100-A10	P60903	*S100A10*	152	35.1	2/6	6.8	5636	5348	5490	5491	83
Protein S100-A11	P31949	*S100A11*	973	34.3	3/43	6.6	26304	94980	30001	50428	22301
Protein S100-A6	P06703	*S100A6*	63	8.9	1/2	5.3	7360	7574	5655	6863	607
Protein-glutamine gamma-glutamyltransferase 2	P21980	*TGM2*	320	16.4	8/13	5.1	34271	36100	28001	32791	2452
Prothymosin alpha	P06454	*PTMA*	87	12.6	2/2	3.7	1708	2465	1973	2049	222
Proto-oncogene serine/threonine-protein kinase mos	P00540	*MOS*	51	2.6	1/15	9.2	118112	86458	107713	104095	9315
Purine nucleoside phosphorylase	P00491	*PNP*	47	12.1	2/4	6.5	4157	6685	5275	5372	731
Putative Ras-related protein Rab-1C	Q92928	*RAB1C*	39	28.9	3/4	5.3	1518	2153	1326	1666	250
Pyruvate dehydrogenase E1 component subunit beta, mitochondrial	P11177	*PDHB*	55	8.9	2/3	6.2	31990	32117	37655	33921	1868
Pyruvate kinase PKM	P14618	*PKM*	2280	55.9	23/173	8.0	397067	567868	467824	477586	49547
Rab GDP dissociation inhibitor beta	P50395	*GDI2*	139	5.6	3/8	6.1	4013	6661	13713	8129	2895
Ras GTPase-activating-like protein IQGAP1	P46940	*IQGAP1*	56	3.7	4/7	6.1	8004	6786	7340	7376	352
Receptor of activated protein C kinase 1	P63244	*RACK1*	42	15.5	4/5	7.6	1002	3333	1849	2061	681
Rho GDP-dissociation inhibitor 1	P52565	*ARHGDIA*	100	7.4	1/2	5.0	4867	3912	1651	3477	954
Rho GDP-dissociation inhibitor 2	P52566	*ARHGDIB*	48	12.9	1/1	5.1	NA	NA	NA	NA	NA
Rho GTPase-activating protein 26	Q9UNA1	*ARHGAP26*	25	2.9	2/17	6.2	NA	NA	NA	NA	NA
Ribosome-binding protein 1	Q9P2E9	*RRBP1*	40	1.9	2/3	8.7	NA	NA	NA	NA	NA
RNA polymerase II-associated protein 1	Q9BWH6	*RPAP1*	32	0.7	1/3	6.0	3437	6928	9264	6543	1693
RNA-binding protein FUS	P35637	*FUS*	231	8.9	3/4	9.4	NA	NA	NA	NA	NA
Serine/threonine-protein phosphatase PP1-gamma catalytic subunit	P36873	*PPP1CC*	45	9.9	2/3	6.1	2873	5623	3209	3902	866
Serpin H1	P50454	*SERPINH1*	125	8.9	2/4	8.8	40068	47929	46178	44725	2383
Signal recognition particle 14 kDa protein	P37108	*SRP14*	100	10.3	1/1	10.1	NA	NA	NA	NA	NA
Small nuclear ribonucleoprotein Sm D1	P62314	*SNRPD1*	138	16.8	1/6	11.6	6398	47268	45270	32978	13303
Splicing factor, proline- and glutamine-rich	P23246	*SFPQ*	94	7.5	4/10	9.5	11573	10818	13601	11998	831
Stathmin	P16949	*STMN1*	54	15.4	2/6	5.8	47794	7520	23965	26426	11691
Stress-70 protein, mitochondrial	P38646	*HSPA9*	47	8.7	5/6	5.9	13151	19381	11846	14792	2325
Stress-induced-phosphoprotein 1	P31948	*STIP1*	45	15.5	6/12	6.4	4714	8504	9411	7543	1439
Sulfotransferase 1A3	P0DMM9	*SULT1A3*	74	11.2	2/4	5.7	NA	NA	NA	NA	NA
Surfeit locus protein 4	O15260	*SURF4*	76	6.7	1/4	7.6	NA	NA	NA	NA	NA
T-complex protein 1 subunit alpha	P17987	*TCP1*	45	9.9	4/6	5.8	4850	10421	13921	9731	2641
T-complex protein 1 subunit delta	P50991	*CCT4*	149	4.8	2/3	8.0	2671	2174	1872	2239	233
T-complex protein 1 subunit eta	Q99832	*CCT7*	234	4.6	1/9	7.6	NA	NA	NA	NA	NA
T-complex protein 1 subunit gamma	P49368	*CCT3*	41	4.8	1/1	6.1	NA	NA	NA	NA	NA
T-complex protein 1 subunit theta	P50990	*CCT8*	109	7.5	3/5	5.4	7786	8308	9644	8579	553
T-complex protein 1 subunit zeta	P40227	*CCT6A*	97	15.6	5/7	6.2	19661	26392	26299	24117	2228
Thioredoxin domain-containing protein 5	Q8NBS9	*TXNDC5*	177	15	5/9	5.6	5551	38511	4363	16142	11190
Transaldolase	P37837	*TALDO1*	41	3.3	1/4	6.4	15741	13947	8625	12771	2137
Transcription elongation factor A protein-like 3	Q969E4	*TCEAL3*	31	24	3/5	4.9	NA	NA	NA	NA	NA
Transcription factor BTF3	P20290	*BTF3*	62	10.7	1/2	9.4	NA	NA	NA	NA	NA
Transgelin-2	P37802	*TAGLN2*	732	49.7	7/28	8.4	57399	110480	76795	81558	15507
Transitional endoplasmic reticulum ATPase	P55072	*VCP*	161	6.8	4/12	5.1	6374	29473	14137	16661	6787
Transketolase	P29401	*TKT*	643	11.4	6/16	7.6	24076	47350	44148	38524	7283
Triosephosphate isomerase	P60174	*TPI1*	643	35.7	7/22	5.7	146216	151248	177844	158436	9812
Tropomyosin alpha-1 chain	P09493	*TPM1*	132	14.1	3/8	4.7	NA	NA	NA	NA	NA
Tropomyosin alpha-3 chain	P06753	*TPM3*	167	9.5	2/6	4.7	11546	13209	13043	12599	529
Tubulin alpha-1A chain	Q71U36	*TUBA1A*	688	31.5	10/34	4.9	NA	NA	NA	NA	NA
Tubulin alpha-1B chain	P68363	*TUBA1B*	708	28.4	9/34	4.9	256115	272977	268453	265848	5039
Tubulin beta chain	P07437	*TUBB*	887	32.7	11/35	4.8	146362	176721	141763	154949	10967
Tubulin beta-4A chain	P04350	*TUBB4A*	396	32.2	9/17	4.8	121377	132544	105460	119794	7859
Tubulin beta-4B chain	P68371	*TUBB4B*	810	32.6	11/33	4.8	NA	NA	NA	NA	NA
Tubulin beta-6 chain	Q9BUF5	*TUBB6*	189	18.8	6/12	4.8	77941	88821	57231	74664	9265
Ubiquitin carboxyl-terminal hydrolase isozyme L1	P09936	*UCHL1*	80	15.2	2/4	5.3	18181	17649	41400	25743	7830
Ubiquitin-40S ribosomal protein S27a	P62979	*RPS27A*	377	19.9	3/10	9.7	NA	NA	NA	NA	NA
Ubiquitin-like modifier-activating enzyme 1	P22314	*UBA1*	335	10.7	7/12	5.5	100384	139631	136812	125609	12639
UPF0183 protein C16orf70	Q9BSU1	*C16orf70*	31	3.6	1/6	7.6	44961	24989	45433	38461	6738
UPF0258 protein KIAA1024	Q9UPX6	*KIAA1024*	38	7.2	5/8	7.0	5248	8423	6191	6621	941
Vimentin	P08670	*VIM*	3540	55.2	28/248	5.1	912676	1042672	935577	963641	40064
Vinculin	P18206	*VCL*	157	6.4	5/11	5.5	23215	25731	31166	26704	2346
X-ray repair cross-complementing protein 6	P12956	*XRCC6*	195	16.4	6/9	6.2	7446	3195	21075	10572	5393
Zyxin	Q15942	*ZYX*	35	2.6	1/1	6.2	4116	1800	1654	2523	797

%Cov = %Sequence coverage = (number of the matched residues/total number of residues in the entire sequence) x 100%.

NA = not applicable (protein was identified in the sample but its MS/MS spectra did not meet predefined criteria for high-confident intensity analysis).

**Table 3:** All proteins identified in EGCG-exposed EA.hy926 endothelial cells.

Protein name	Swiss-Prot ID	Gene symbol	MS/MS identification score	% Cov	No. of distinct/total matched peptides	p*I*	Intensity (x10^3^ arbitrary unit)				
							N1	N2	N3	Mean	SEM
14-3-3 protein beta/alpha	P31946	*YWHAB*	142	12.2	2/7	4.8	14856	25768	24104	21576	3394
14-3-3 protein epsilon	P62258	*YWHAE*	126	24.3	4/6	4.6	9737	11989	6523	9416	1586
14-3-3 protein theta	P27348	*YWHAQ*	75	15.5	4/4	4.7	35166	33504	30882	33184	1247
14-3-3 protein zeta/delta	P63104	*YWHAZ*	150	45.3	9/20	4.7	23094	28757	41849	31234	5554
26S protease regulatory subunit 6A	P17980	*PSMC3*	88	11.4	3/5	5.1	17797	51266	42507	37190	10021
26S protease regulatory subunit 6B	P43686	*PSMC4*	47	5.3	3/5	5.1	94361	84996	70360	83239	6984
26S protease regulatory subunit 7	P35998	*PSMC2*	79	5.8	2/4	5.7	17425	17155	14886	16489	805
26S proteasome non-ATPase regulatory subunit 12	O00232	*PSMD12*	27	8.3	3/9	7.5	32563	32999	27461	31007	1778
40S ribosomal protein S11	P62280	*RPS11*	61	7	1/2	10.3	2520	4150	5421	4030	839
40S ribosomal protein S12	P25398	*RPS12*	86	34.1	4/7	6.8	109186	7943	7527	41552	33817
40S ribosomal protein S13	P62277	*RPS13*	150	17.9	2/8	10.5	10185	8264	7963	8804	696
40S ribosomal protein S14	P62263	*RPS14*	103	7.3	1/2	10.1	18814	9167	2434	10138	4753
40S ribosomal protein S15	P62841	*RPS15*	94	13.1	1/3	10.4	16183	18812	4042	13012	4549
40S ribosomal protein S16	P62249	*RPS16*	177	21.9	3/6	10.2	34980	27926	27421	30109	2440
40S ribosomal protein S18	P62269	*RPS18*	47	5.3	1/2	11.0	5469	3885	10012	6455	1836
40S ribosomal protein S19	P39019	*RPS19*	77	25.5	4/4	10.3	41636	62034	41193	48288	6874
40S ribosomal protein S2	P15880	*RPS2*	84	19.5	4/6	10.3	70528	78705	69344	72859	2943
40S ribosomal protein S20	P60866	*RPS20*	60	10.1	1/4	10.0	12786	10273	18891	13983	2559
40S ribosomal protein S24	P62847	*RPS24*	34	11.3	1/3	10.8	4519	2670	1627	2939	846
40S ribosomal protein S25	P62851	*RPS25*	85	8	1/2	10.1	6522	2931	7305	5586	1347
40S ribosomal protein S3	P23396	*RPS3*	49	10.7	2/3	9.7	4583	6576	5298	5485	583
40S ribosomal protein S30	P62861	*FAU*	49	16.9	1/2	12.2	9426	6156	10466	8683	1299
40S ribosomal protein S3a	P61247	*RPS3A*	66	11	3/6	9.8	8707	7249	5894	7283	812
40S ribosomal protein S5	P46782	*RPS5*	717	7.4	1/12	9.7	99724	82871	71606	84734	8170
40S ribosomal protein S7	P62081	*RPS7*	114	26.8	5/9	10.1	4548	11324	9732	8535	2045
40S ribosomal protein S8	P62241	*RPS8*	62	10.6	2/4	10.3	4813	6137	7097	6016	662
40S ribosomal protein SA	P08865	*RPSA*	111	9.5	2/4	4.8	11151	8777	13306	11078	1308
60 kDa heat shock protein, mitochondrial	P10809	*HSPD1*	869	19.2	9/30	5.7	82898	58620	65020	68846	7265
60S acidic ribosomal protein P0-like	Q8NHW5	*RPLP0P6*	78	17.7	4/5	5.4	NA	NA	NA	NA	NA
60S acidic ribosomal protein P1	P05386	*RPLP1*	39	14	1/2	4.3	53323	21160	38947	37810	9302
60S acidic ribosomal protein P2	P05387	*RPLP2*	66	39.1	2/3	4.4	9083	20753	6118	11985	4467
60S ribosomal protein L10	P27635	*RPL10*	111	6.5	1/3	10.1	864	1808	7593	3422	2104
60S ribosomal protein L12	P30050	*RPL12*	131	28.5	3/5	9.5	9704	14112	7536	10451	1935
60S ribosomal protein L15	P61313	*RPL15*	39	4.4	1/1	11.6	6040	1241	5128	4137	1471
60S ribosomal protein L18	Q07020	*RPL18*	98	6.9	1/4	11.7	7596	8074	5352	7007	839
60S ribosomal protein L19	P84098	*RPL19*	62	4.6	1/2	11.5	22554	1900	21706	15387	6748
60S ribosomal protein L23	P62829	*RPL23*	95	14.3	1/2	10.5	2755	6535	10270	6520	2169
60S ribosomal protein L27	P61353	*RPL27*	35	6.6	1/1	10.6	2976	9151	4588	5572	1849
60S ribosomal protein L27a	P46776	*RPL27A*	49	11.5	2/4	11.0	26212	27037	17693	23647	2987
60S ribosomal protein L29	P47914	*RPL29*	77	9.4	1/2	11.7	5297	4889	8918	6368	1280
60S ribosomal protein L30	P62888	*RPL30*	95	13.9	1/2	9.7	27044	23235	8346	19542	5705
60S ribosomal protein L36	Q9Y3U8	*RPL36*	41	8.6	1/1	11.6	5046	3454	2748	3749	680
60S ribosomal protein L38	P63173	*RPL38*	92	35.7	2/8	10.1	NA	NA	NA	NA	NA
60S ribosomal protein L4	P36578	*RPL4*	43	2.8	1/1	11.1	10869	10149	5538	8852	1670
60S ribosomal protein L6	Q02878	*RPL6*	54	6.3	2/4	10.6	14176	6382	3399	7986	3213
60S ribosomal protein L7	P18124	*RPL7*	104	17.3	4/5	10.7	11702	7014	6816	8511	1597
60S ribosomal protein L7a	P62424	*RPL7A*	71	17.7	5/7	10.6	14133	9188	7774	10365	1928
78 kDa glucose-regulated protein	P11021	*HSPA5*	1086	27.8	16/40	5.1	NA	NA	NA	NA	NA
Acidic leucine-rich nuclear phosphoprotein 32 family member A	P39687	*ANP32A*	68	8.4	1/4	4.0	NA	NA	NA	NA	NA
Actin, aortic smooth muscle	P62736	*ACTA2*	1823	23.1	9/163	5.2	NA	NA	NA	NA	NA
Activator of 90 kDa heat shock protein ATPase homolog 1	O95433	*AHSA1*	96	21.6	4/5	5.4	NA	NA	NA	NA	NA
ADP/ATP translocase 2	P05141	*SLC25A5*	64	13.1	4/7	9.7	46370	18671	43459	36167	8788
ADP-ribosylation factor 1	P84077	*ARF1*	30	9.9	1/2	6.3	NA	NA	NA	NA	NA
Alpha-actinin-1	P12814	*ACTN1*	149	9.9	8/13	5.3	NA	NA	NA	NA	NA
Alpha-actinin-4	O43707	*ACTN4*	272	13.4	8/11	5.3	90204	96621	81692	89506	4324
Alpha-enolase	P06733	*ENO1*	1868	45.2	13/419	7.0	202358	212994	130496	181949	25909
Annexin A1	P04083	*ANXA1*	1066	45.1	12/35	6.6	148786	110784	108081	122551	13141
Annexin A2	P07355	*ANXA2*	1653	59.3	19/73	7.6	NA	NA	NA	NA	NA
Annexin A5	P08758	*ANXA5*	1475	41.3	13/44	4.9	475467	433887	276964	395440	60441
ATP synthase subunit alpha, mitochondrial	P25705	*ATP5F1A*	310	5.8	2/6	9.2	83468	99810	108357	97211	7302
ATP synthase subunit beta, mitochondrial	P06576	*ATP5F1B*	429	27	10/21	5.3	48637	37303	23580	36507	7244
ATP-dependent RNA helicase A	Q08211	*DHX9*	32	3.5	4/9	6.4	1931	4245	3151	3109	668
ATP-dependent RNA helicase DDX3X	O00571	*DDX3X*	48	7.4	3/6	6.7	NA	NA	NA	NA	NA
Barrier-to-autointegration factor	O75531	*BANF1*	67	27	1/2	5.8	NA	NA	NA	NA	NA
Basic leucine zipper and W2 domain-containing protein 1	Q7L1Q6	*BZW1*	171	8.6	3/4	5.8	NA	NA	NA	NA	NA
Beta-actin-like protein 2	Q562R1	*ACTBL2*	1238	17.6	6/237	5.4	352799	135984	323943	270909	67975
Beta-enolase	P13929	*ENO3*	564	21.7	5/80	7.6	NA	NA	NA	NA	NA
Calnexin	P27824	*CANX*	130	7.1	3/5	4.5	NA	NA	NA	NA	NA
Calpain small subunit 1	P04632	*CAPNS1*	50	9	1/2	5.1	NA	NA	NA	NA	NA
Calreticulin	P27797	*CALR*	96	12	3/6	4.3	2845	5237	8126	5403	1527
Cathepsin D	P07339	*CTSD*	86	10.7	3/6	6.1	33499	28185	24919	28867	2500
Caveolin-1	Q03135	*CAV1*	87	13.5	2/3	5.7	NA	NA	NA	NA	NA
CD59 glycoprotein	P13987	*CD59*	28	9.4	1/1	6.0	NA	NA	NA	NA	NA
Centriolin	Q7Z7A1	*CNTRL*	30	2.8	7/13	5.4	10908	14867	3245	9673	3411
Chloride intracellular channel protein 1	O00299	*CLIC1*	152	12.4	2/3	5.1	30494	32869	20242	27868	3874
Chloride intracellular channel protein 4	Q9Y696	*CLIC4*	50	16.6	3/6	5.5	NA	NA	NA	NA	NA
Clathrin heavy chain 1	Q00610	*CLTC*	114	4.5	5/9	5.5	NA	NA	NA	NA	NA
Coatomer subunit gamma-1	Q9Y678	*COPG1*	64	3.7	2/2	5.3	NA	NA	NA	NA	NA
Cofilin-1	P23528	*RPS3*	78	6.6	1/2	8.2	20964	16535	14004	17168	2034
Cofilin-2	Q9Y281	*CFL2*	70	11.4	2/3	7.7	NA	NA	NA	NA	NA
Copine-1	Q99829	*CPNE1*	136	10.2	4/31	5.5	12777	9149	13899	11941	1433
CREB-binding protein	Q92793	*CREBBP*	30	0.5	1/2	8.8	3965	3520	7964	5150	1413
Cysteine and glycine-rich protein 1	P21291	*CSRP1*	40	8.8	1/2	8.9	NA	NA	NA	NA	NA
Cytoplasmic dynein 1 heavy chain 1	Q14204	*DYNC1H1*	31	2.8	10/12	6.0	NA	NA	NA	NA	NA
Cytoskeleton-associated protein 4	Q07065	*CKAP4*	328	25.7	11/19	5.6	56769	66405	59821	60998	2843
Dedicator of cytokinesis protein 10	Q96BY6	*DOCK10*	29	2	4/10	6.7	28536	33353	18905	26932	4247
Dehydrogenase/reductase SDR family member 12	A0PJE2	*DHRS12*	38	2.5	1/1	6.8	NA	NA	NA	NA	NA
DNA damage-binding protein 1	Q16531	*DDB1*	136	3.4	3/5	5.1	NA	NA	NA	NA	NA
DNA-(apurinic or apyrimidinic site) lyase	P27695	*APEX1*	54	5.3	1/2	8.3	6105	4535	4671	5104	502
EH domain-containing protein 1	Q9H4M9	*EHD1*	42	7.9	2/2	6.4	4883	3519	3475	3959	462
Elongation factor 1-alpha 1	P68104	*EEF1A1*	1873	27.1	7/337	9.1	61754	77139	66647	68513	4538
Elongation factor 1-delta	P29692	*EEF1D*	118	16.7	4/6	4.9	5820	6117	4248	5395	580
Elongation factor 1-gamma	P26641	*EEF1G*	40	9.2	5/7	6.3	21454	8287	8522	12755	4350
Elongation factor 2	P13639	*EEF2*	782	24.4	13/34	6.4	273816	181870	119469	191719	44827
Elongation factor Tu, mitochondrial	P49411	*TUFM*	104	15.3	4/6	7.3	100052	121532	94084	105223	8335
Endoplasmin	P14625	*HSP90B1*	384	10.5	7/17	4.8	NA	NA	NA	NA	NA
Enoyl-CoA hydratase, mitochondrial	P30084	*ECHS1*	126	5.9	1/2	8.3	19025	6669	4936	10210	4436
Eukaryotic initiation factor 4A-I	P60842	*EIF4A1*	186	8.6	3/7	5.3	15563	6869	21492	14641	4246
Eukaryotic translation initiation factor 3 subunit E	P60228	*EIF3E*	76	4.3	1/2	5.7	NA	NA	NA	NA	NA
Eukaryotic translation initiation factor 3 subunit H	O15372	*EIF3H*	31	5.4	1/1	6.1	986	3977	1907	2290	884
Eukaryotic translation initiation factor 4B	P23588	*EIF4B*	53	2.8	1/2	5.6	NA	NA	NA	NA	NA
Eukaryotic translation initiation factor 5A-1	P63241	*EIF5A*	59	5.2	1/1	5.1	5721	3751	4992	4821	575
Exportin-2	P55060	*CSE1L*	39	3.5	3/239	5.5	162139	124377	170237	152251	14132
Ezrin	P15311	*EZR*	293	12.1	8/16	5.9	18275	28096	44421	30264	7625
FACT complex subunit SSRP1	Q08945	*SSRP1*	65	6.5	3/7	6.5	NA	NA	NA	NA	NA
F-actin-capping protein subunit alpha-1	P52907	*CAPZA1*	47	6.3	1/2	5.5	NA	NA	NA	NA	NA
F-actin-capping protein subunit beta	P47756	*CAPZB*	49	18.4	3/6	5.4	NA	NA	NA	NA	NA
Fascin	Q16658	*FSCN1*	44	8.5	3/3	6.8	NA	NA	NA	NA	NA
F-box/LRR-repeat protein 19	Q6PCT2	*FBXL19*	30	2	2/3	9.4	13989	14989	6940	11973	2533
Filamin-A	P21333	*FLNA*	534	12.6	21/38	5.7	125578	212121	129322	155673	28244
Filamin-B	O75369	*FLNB*	129	5.5	10/14	5.5	46259	120035	47982	71425	24310
Fructose-bisphosphate aldolase A	P04075	*ALDOA*	477	34.9	10/24	8.3	55206	76813	42632	58217	9981
Fructose-bisphosphate aldolase C	P09972	*ALDOC*	35	6.3	1/1	6.4	5871	3760	2508	4046	981
Galectin-1	P09382	*LGALS1*	376	40.7	5/14	5.3	53517	62833	55917	57422	2793
Glucose-6-phosphate isomerase	P06744	*GPI*	120	7.2	2/5	8.4	115873	121211	95298	110794	7900
Glucosidase 2 subunit beta	P14314	*PRKCSH*	70	8	4/6	4.3	41191	66489	26569	44750	11660
Glutathione S-transferase P	P09211	*GSTP1*	310	41.9	4/12	5.4	12223	11704	11259	11729	278
Glyceraldehyde-3-phosphate dehydrogenase	P04406	*GAPDH*	6498	54.6	14/564	8.6	566470	555632	527936	550012	11473
Glycerol kinase 2	Q14410	*GK2*	49	1.6	1/4	5.6	NA	NA	NA	NA	NA
GTP-binding nuclear protein Ran	P62826	*RAN*	121	15.7	3/6	7.0	52227	53052	55041	53440	835
Heat shock 70 kDa protein 6	P17066	*HSPA6*	335	8.9	5/10	5.8	77096	77483	54634	69738	7553
Heat shock cognate 71 kDa protein	P11142	*HSPA8*	946	26.2	16/40	5.4	NA	NA	NA	NA	NA
Heat shock protein beta-1	P04792	*HSPB1*	204	38.5	7/13	6.0	111538	89278	84258	95025	8383
Heat shock protein HSP 90-alpha	P07900	*HSP90AA1*	756	20.5	13/36	4.9	251797	298927	299546	283423	15814
Heat shock protein HSP 90-beta	P08238	*HSP90AB1*	771	25.7	16/44	5.0	192504	242048	217251	217268	14302
Hemoglobin subunit alpha	P69905	*HBA1*	65	10.6	1/3	8.7	5858	9805	8328	7997	1151
Heterogeneous nuclear ribonucleoprotein A1	P09651	*HNRNPA1*	180	25.5	6/8	9.2	27144	24090	23505	24913	1128
Heterogeneous nuclear ribonucleoprotein A3	P51991	*HNRNPA3*	54	3.4	1/2	9.1	NA	NA	NA	NA	NA
Heterogeneous nuclear ribonucleoprotein D0	Q14103	*HNRNPD*	37	12.4	3/4	7.6	8847	7827	12568	9748	1441
Heterogeneous nuclear ribonucleoprotein H	P31943	*HNRNPH1*	256	12.5	4/11	5.9	26946	25624	23427	25333	1026
Heterogeneous nuclear ribonucleoprotein H3	P31942	*HNRNPH3*	139	4.9	1/2	6.4	6109	6304	3023	5145	1062
Heterogeneous nuclear ribonucleoprotein K	P61978	*HNRNPK*	146	19.7	7/10	5.4	38622	17556	17898	24692	6966
Heterogeneous nuclear ribonucleoprotein M	P52272	*HNRNPM*	59	12.3	7/8	8.8	5758	21460	9153	12124	4770
Heterogeneous nuclear ribonucleoprotein Q	O60506	*SYNCRIP*	65	4.2	2/6	8.7	13745	23603	21745	19698	3024
Heterogeneous nuclear ribonucleoprotein R	O43390	*HNRNPR*	76	4.1	2/3	8.2	9155	10674	8635	9488	612
Heterogeneous nuclear ribonucleoprotein U	Q00839	*HNRNPU*	159	9.9	6/10	5.8	5461	10594	6729	7594	1544
Heterogeneous nuclear ribonucleoproteins A2/B1	P22626	*HNRNPA2B1*	231	14.7	4/19	9.0	13444	15814	31249	20169	5582
Heterogeneous nuclear ribonucleoproteins C1/C2	P07910	*HNRNPC*	66	5.9	2/5	5.0	NA	NA	NA	NA	NA
Histone H1.0	P07305	*H1F0*	38	8.2	2/2	10.8	5480	3885	10012	6459	1835
Histone H1.3	P16402	*HIST1H1D*	258	15.8	4/11	11.0	NA	NA	NA	NA	NA
Histone H1.4	P10412	*HIST1H1E*	251	21.5	4/8	11.0	30814	21041	47941	33265	7862
Histone H1.5	P16401	*HIST1H1B*	56	6.6	2/3	10.9	41092	43699	38926	41239	1380
Histone H2A type 1-B/E	P04908	*HIST1H2AB*	807	23.1	2/14	11.1	226485	254958	230893	237445	8848
Histone H2A type 1-D	P20671	*HIST1H2AD*	962	23.1	2/18	10.9	NA	NA	NA	NA	NA
Histone H2B type 1-B	P33778	*HIST1H2BB*	3114	41.3	6/528	10.3	NA	NA	NA	NA	NA
Histone H2B type 1-C/E/F/G/I	P62807	*HIST1H2BC*	2764	35.7	5/97	10.3	NA	NA	NA	NA	NA
Histone H2B type 1-M	Q99879	*HIST1H2BM*	3154	46	7/565	10.3	NA	NA	NA	NA	NA
Histone H3.1t	Q16695	*HIST3H3*	218	23.5	5/18	11.1	NA	NA	NA	NA	NA
Histone H4	P62805	*HIST1H4A*	1618	55.3	8/193	11.4	434182	394339	417448	415323	11550
Importin subunit beta-1	Q14974	*KPNB1*	54	6.1	4/5	4.7	15112	13386	11843	13447	944
Importin-9	Q96P70	*IPO9*	28	2.7	1/1	4.7	NA	NA	NA	NA	NA
Inosine-5′-monophosphate dehydrogenase 2	P12268	*IMPDH2*	56	4.5	2/3	6.4	7785	13828	8262	9958	1940
Interleukin enhancer-binding factor 3	Q12906	*ILF3*	74	5.1	3/4	8.9	3331	2907	5355	3864	755
Kelch-like protein 35	Q6PF15	*KLHL35*	44	1.2	1/32	8.1	124565	146490	86916	119324	17396
Keratin, type I cytoskeletal 18	P05783	*KRT18*	468	37.4	11/22	5.3	124666	108627	93075	108789	9120
Keratin, type II cytoskeletal 1b	Q7Z794	*KRT77*	39	6.6	2/11	5.7	5480	3885	10012	6459	1835
Keratin, type II cytoskeletal 7	P08729	*KRT7*	495	26.2	11/27	5.4	119134	129076	105812	118007	6739
Keratin, type II cytoskeletal 8	P05787	*KRT8*	651	36.6	17/39	5.5	185098	187010	191185	187764	1797
Laminin subunit beta-1	P07942	*LAMB1*	35	3.5	5/7	4.8	NA	NA	NA	NA	NA
l-lactate dehydrogenase A chain	P00338	*LDHA*	343	25.9	8/16	8.4	108915	111146	97752	105938	4143
l-lactate dehydrogenase B chain	P07195	*LDHB*	807	24.3	7/21	5.7	170067	174272	155237	166525	5773
Lysine-specific demethylase 2B	Q8NHM5	*KDM2B*	39	1.8	3/4	8.9	NA	NA	NA	NA	NA
Macrophage migration inhibitory factor	P14174	*MIF*	45	30.4	2/3	7.7	11233	6627	11216	9692	1532
Malignant T-cell-amplified sequence 1	Q9ULC4	*MCTS1*	73	9.4	1/3	9.0	20802	17649	15260	17904	1605
Moesin	P26038	*MSN*	910	30.8	16/45	6.1	144752	101440	138689	128294	13540
Myosin light polypeptide 6	P60660	*MYL6*	155	29.1	3/6	4.6	NA	NA	NA	NA	NA
Myosin-9	P35579	*MYH9*	497	13.2	17/51	5.5	149595	188171	197928	178565	14756
Myristoylated alanine-rich C-kinase substrate	P29966	*MARCKS*	99	6.9	1/2	4.5	NA	NA	NA	NA	NA
Nascent polypeptide-associated complex subunit alpha, muscle-specific form	E9PAV3	*NACA*	140	2.5	3/5	9.6	19674	4499	5443	9872	4908
Neuroblast differentiation-associated protein AHNAK	Q09666	*AHNAK*	55	3.7	18/33	5.8	11644	14467	15574	13895	1170
Non-POU domain-containing octamer-binding protein	Q15233	*NONO*	40	4.9	1/2	9.0	NA	NA	NA	NA	NA
Nuclear autoantigenic sperm protein	P49321	*NASP*	25	2.9	1/1	4.3	NA	NA	NA	NA	NA
Nucleolin	P19338	*NCL*	334	19.9	9/25	4.6	5066	3357	3897	4107	504
Nucleophosmin	P06748	*NPM1*	633	25.5	6/23	4.6	84527	48445	63459	65477	10465
Nucleoside diphosphate kinase A	P15531	*NME1*	182	28.9	3/14	5.8	NA	NA	NA	NA	NA
Nucleosome assembly protein 1-like 1	P55209	*NAP1L1*	208	9	2/5	4.4	14940	17948	17631	16840	954
Obg-like ATPase 1	Q9NTK5	*OLA1*	56	7.1	2/3	7.6	13317	6615	5783	8572	2385
Parathymosin	P20962	*PTMS*	85	11.8	1/2	4.1	NA	NA	NA	NA	NA
Peptidyl-prolyl *cis*-trans isomerase A	P62937	*PPIA*	926	58.2	9/46	7.7	334903	328382	274086	312457	19278
Peptidyl-prolyl *cis*-trans isomerase B	P23284	*PPIB*	227	22.7	4/8	9.4	52652	43189	45463	47101	2852
Peroxiredoxin-1	Q06830	*PRDX1*	167	49.2	10/18	8.3	41901	37568	40305	39925	1265
Peroxiredoxin-6	P30041	*PRDX6*	50	12.5	2/3	6.0	2427	2418	2925	2590	168
Phosphoglycerate kinase 1	P00558	*PGK1*	362	26.9	8/17	8.3	42360	48337	45011	45236	1729
Phosphoglycerate mutase 1	P18669	*PGAM1*	147	17.7	3/8	6.7	NA	NA	NA	NA	NA
Plasminogen activator inhibitor 2	P05120	*SERPINB2*	48	6.7	2/3	5.5	28677	19043	12147	19956	4793
Plastin-3	P13797	*PLS3*	107	12.4	5/13	5.4	46429	52915	66457	55267	5900
Plectin	Q15149	*PLEC*	512	11.9	45/72	5.7	335859	212029	106429	218106	66300
Poly(rC)-binding protein 2	Q15366	*PCBP2*	131	14.2	4/7	6.3	NA	NA	NA	NA	NA
Polymerase I and transcript release factor	Q6NZI2	*CAVIN1*	205	9	3/5	5.5	31186	37049	22373	30203	4265
Polypyrimidine tract-binding protein 1	P26599	*PTBP1*	37	3.8	1/2	9.2	NA	NA	NA	NA	NA
PRA1 family protein 3	O75915	*ARL6IP5*	102	14.9	2/3	9.8	NA	NA	NA	NA	NA
Prelamin-A/C	P02545	*LMNA*	756	29.4	16/31	6.6	199768	214589	195265	203207	5837
Probable ATP-dependent RNA helicase DDX17	Q92841	*DDX17*	42	8.2	5/6	8.5	NA	NA	NA	NA	NA
Probable ATP-dependent RNA helicase DDX5	P17844	*DDX5*	102	4.4	2/4	9.1	70159	72621	63348	68709	2773
Profilin-1	P07737	*PFN1*	158	61.4	8/13	8.4	52591	56414	50850	53285	1643
Prohibitin	P35232	*PHB*	32	3.7	1/4	5.6	7468	7390	4636	6498	931
Prohibitin-2	Q99623	*PHB2*	40	4	1/1	9.8	2140	4504	3240	3295	683
Proliferating cell nuclear antigen	P12004	*PCNA*	41	16.9	3/4	4.6	12659	20827	15557	16348	2391
Proliferation-associated protein 2G4	Q9UQ80	*PA2G4*	55	6.3	2/3	6.1	10199	14511	10920	11877	1333
Proteasome activator complex subunit 2	Q9UL46	*PSME2*	85	5.4	1/2	5.5	11756	6448	7849	8684	1588
Protein disulfide-isomerase A3	P30101	*PDIA3*	160	17	6/9	6.0	23578	27498	24887	25321	1152
Protein disulfide-isomerase A4	P13667	*PDIA4*	59	3.4	2/3	5.0	14875	5481	10585	10314	2715
Protein disulfide-isomerase A6	Q15084	*PDIA6*	176	16.4	4/8	5.0	35284	43729	28531	35848	4396
Protein S100-A10	P60903	*S100A10*	101	45.4	3/6	6.8	NA	NA	NA	NA	NA
Protein S100-A11	P31949	*S100A11*	1157	34.3	3/41	6.6	98925	46580	67624	71043	15207
Protein S100-A6	P06703	*S100A6*	65	16.7	2/4	5.3	14068	16731	7122	12641	2864
Protein transport protein Sec61 subunit beta	P60468	*SEC61B*	40	20.8	2/2	11.6	NA	NA	NA	NA	NA
Protein-glutamine gamma-glutamyltransferase 2	P21980	*TGM2*	280	16.4	8/12	5.1	42175	39248	42729	41384	1080
Prothymosin alpha	P06454	*PTMA*	125	12.6	1/2	3.7	NA	NA	NA	NA	NA
Proto-oncogene serine/threonine-protein kinase mos	P00540	*MOS*	42	6.4	2/235	9.2	162044	123745	169678	151822	14211
Pyruvate dehydrogenase E1 component subunit beta, mitochondrial	P11177	*PDHB*	68	7.5	2/3	6.2	50008	38284	38838	42377	3819
Pyruvate kinase PKM	P14618	*PKM*	1981	56.9	24/145	8.0	541639	405570	446687	464632	40291
Rab GDP dissociation inhibitor beta	P50395	*GDI2*	85	4.9	2/7	6.1	14841	13465	10350	12886	1329
Ras-related C3 botulinum toxin substrate 1	P63000	*RAC1*	45	15.6	3/4	8.8	NA	NA	NA	NA	NA
Ras-related protein Rab-10	P61026	*RAB10*	84	20	3/4	8.6	NA	NA	NA	NA	NA
Ras-related protein Rab-1B	Q9H0U4	*RAB1B*	37	21.4	3/5	5.6	NA	NA	NA	NA	NA
Ras-related protein Rab-7a	P51149	*RAB7A*	41	6.8	1/2	6.4	9007	10021	8229	9086	519
Receptor of activated protein C kinase 1	P63244	*RACK1*	40	4.4	2/3	7.6	1669	8683	9471	6608	2480
Rho GDP-dissociation inhibitor 1	P52565	*ARHGDIA*	113	7.4	1/2	5.0	7234	3657	4415	5102	1088
Ribosome-binding protein 1	Q9P2E9	*RRBP1*	41	4.4	5/6	8.7	NA	NA	NA	NA	NA
RNA-binding protein FUS	P35637	*FUS*	254	7.8	2/6	9.4	NA	NA	NA	NA	NA
Serine/threonine-protein phosphatase PP1-gamma catalytic subunit	P36873	*PPP1CC*	48	14.2	3/5	6.1	5720	13195	15886	11600	3041
Serpin H1	P50454	*SERPINH1*	139	14.4	3/5	8.8	8221	28883	39811	25638	9262
Signal recognition particle 14 kDa protein	P37108	*SRP14*	171	10.3	1/2	10.1	NA	NA	NA	NA	NA
Small nuclear ribonucleoprotein Sm D1	P62314	*SNRPD1*	75	16.8	1/5	11.6	36616	14165	13818	21533	7542
Stress-70 protein, mitochondrial	P38646	*HSPA9*	61	10.8	6/9	5.9	42451	53345	45465	47087	3248
Sulfotransferase 1A3	P0DMM9	*SULT1A3*	83	11.2	2/4	5.7	NA	NA	NA	NA	NA
Surfeit locus protein 4	O15260	*SURF4*	113	6.7	1/4	7.6	NA	NA	NA	NA	NA
T-complex protein 1 subunit alpha	P17987	*TCP1*	31	8.1	3/3	5.8	NA	NA	NA	NA	NA
T-complex protein 1 subunit delta	P50991	*CCT4*	133	5.9	3/4	8.0	2861	3278	3014	3051	122
T-complex protein 1 subunit eta	Q99832	*CCT7*	219	8.7	2/9	7.6	NA	NA	NA	NA	NA
T-complex protein 1 subunit gamma	P49368	*CCT3*	36	11	3/3	6.1	NA	NA	NA	NA	NA
T-complex protein 1 subunit theta	P50990	*CCT8*	107	5.8	2/4	5.4	6878	3385	19274	9846	4821
T-complex protein 1 subunit zeta	P40227	*CCT6A*	47	11.7	5/8	6.2	22142	22782	12656	19194	3274
Thioredoxin domain-containing protein 5	Q8NBS9	*TXNDC5*	206	13	4/6	5.6	4738	41071	7478	17763	11681
Tight junction protein ZO-1	Q07157	*TJP1*	27	3.7	5/8	6.2	NA	NA	NA	NA	NA
Transaldolase	P37837	*TALDO1*	31	4.7	2/4	6.4	14850	6899	7830	9860	2509
Transcription elongation factor A protein-like 3	Q969E4	*TCEAL3*	46	20	2/3	4.9	NA	NA	NA	NA	NA
Transcription factor BTF3	P20290	*BTF3*	67	24.8	2/3	9.4	NA	NA	NA	NA	NA
Transgelin-2	P37802	*TAGLN2*	605	52.8	8/26	8.4	96536	82842	76076	85151	6018
Transitional endoplasmic reticulum ATPase	P55072	*VCP*	336	10.7	6/17	5.1	48158	25146	19881	31062	8682
Transketolase	P29401	*TKT*	611	13	7/22	7.6	62407	56349	45118	54624	5065
Triosephosphate isomerase	P60174	*TPI1*	623	40.6	8/24	5.7	161006	184029	208480	184505	13707
Tropomodulin-3	Q9NYL9	*TMOD3*	56	9.7	2/2	5.1	3612	2492	3531	3212	361
Tropomyosin alpha-1 chain	P09493	*TPM1*	116	15.1	3/6	4.7	NA	NA	NA	NA	NA
Tropomyosin alpha-4 chain	P67936	*TPM4*	156	18.5	4/7	4.7	NA	NA	NA	NA	NA
Tubulin alpha-1B chain	P68363	*TUBA1B*	730	32.8	10/33	4.9	253775	282977	235455	257402	13838
Tubulin beta chain	P07437	*TUBB*	969	34.2	11/36	4.8	202837	201022	190264	198041	3924
Tubulin beta-4B chain	P68371	*TUBB4B*	929	34.2	11/34	4.8	NA	NA	NA	NA	NA
Tubulin beta-6 chain	Q9BUF5	*TUBB6*	223	24.2	8/19	4.8	98391	92260	91928	94193	2101
Ubiquinone biosynthesis monooxygenase COQ6, mitochondrial	Q9Y2Z9	*COQ6*	35	1.9	1/234	6.8	162139	124377	170237	152251	14132
Ubiquitin carboxyl-terminal hydrolase isozyme L1	P09936	*UCHL1*	58	22	3/5	5.3	13815	12083	9694	11864	1195
Ubiquitin-40S ribosomal protein S27a	P62979	*RPS27A*	334	25.6	4/13	9.7	NA	NA	NA	NA	NA
Ubiquitin-like modifier-activating enzyme 1	P22314	*UBA1*	195	8.3	5/12	5.5	94700	122097	107087	107961	7921
UPF0258 protein KIAA1024	Q9UPX6	*KIAA1024*	30	4	3/4	7.0	9236	5429	3319	5995	1731
Vimentin	P08670	*VIM*	3693	59.4	29/320	5.1	916825	966574	861243	914880	30422
Vinculin	P18206	*VCL*	100	8.9	8/12	5.5	22380	22607	10146	18378	4116
Voltage-dependent anion-selective channel protein 1	P21796	*VDAC1*	65	17.3	4/6	8.6	18338	13894	3640	11957	4352
X-ray repair cross-complementing protein 6	P12956	*XRCC6*	232	13.5	5/11	6.2	12431	22365	7683	14160	4326
Zyxin	Q15942	*ZYX*	32	2.1	1/1	6.2	16040	8804	16016	13620	2408

%Cov = %Sequence coverage = (number of the matched residues/total number of residues in the entire sequence) x 100%.

NA = not applicable (protein was identified in the sample but its MS/MS spectra did not meet predefined criteria for high-confident intensity analysis).

## 2 Experimental design, materials, and methods

### 2.1 Cell culture

Human endothelial cell line (EA.hy926) (ATCC; Manassas, VA) was grown and maintained in a complete medium (DMEM/F12) (Gibco, Invitrogen; Grand Island, NY), supplemented with 10% heat-inactivated fetal bovine serum (FBS), 60 U/ml penicillin G and 60 μg/ml streptomycin, in a humidified incubator with 5% CO_2_ at 37 °C.

### 2.2 Treatment with caffeine and EGCG

EA.hy926 cells (from the 5th passage) were seeded in 6-well plate (Corning Inc.; Corning, NY) at a density of 5 × 10^5^ cells/well and maintained in the complete medium for 24-h prior to experiment. The cells were treated with 100 μM caffeine or EGCG (both were from Sigma-Aldrich; St. Louis, MO) for 24-h, whereas the untreated cells served as the control (n = 3 independent biological replicates per group; a total of 9 biological samples were analyzed). Thereafter, the cell monolayers were washed with PBS five times and then scraped. After collecting cell pellets by centrifugation at 1000 *g*, cellular proteins were extracted using SDT lysis buffer (4% SDS, 100 mM DTT, and 100 mM Tris-HCl; pH 7.6). Protein concentrations were quantified by Bradford's method using Bio-Rad protein assay (Bio-Rad; Milano, Italy).

### 2.3 In-solution tryptic digestion by filter-aided sample preparation (FASP) method

In-solution tryptic digestion was performed as described previously [2], [3]. Briefly, protein samples prepared in SDT lysis buffer were reduced by heating at 95 °C for 5 min. After cooling down at 25 °C, an equal amount of protein (30 μg/sample) was transferred to an Omega Nanosep 10K device (Pall Corporation; Port Washington, NY), added with 200 μl of 8 M urea in 100 mM Tris-HCl (pH 8.5), and then centrifuged at 14,000 *g* and 25 °C for 15 min. This buffer exchange step was repeated one more cycle. The recovered proteins were then alkylated with 100 μl of 50 mM iodoacetamide in 8 M urea/100 mM Tris-HCl (pH 8.5) at 25 °C in the dark using a ThermoMixer^®^ C (Eppendorf; Hauppauge, NY) for 20 min. Thereafter, buffer exchange was performed twice by centrifugation at 14,000 *g* and 25 °C for 15 min each using 200 μl of 8 M urea/100 mM Tris-HCl (pH 8.5). The proteins were then finally exchanged into 50 mM NH_4_HCO_3_ and digested with sequencing grade modified trypsin (Promega; Madison, WI) in 50 mM NH_4_HCO_3_ at a ratio of 1:50 (w/w) trypsin/protein at 37 °C for 16–18 h in a ThermoMixer^®^ C. The digested peptides were collected by transferring the filter unit to a new collection tube and centrifuged at 14,000 *g* and 25 °C for 10–20 min. Trypsin activity was then stopped by adding 10 μl of 5% formic acid in 80% acetronitrile (ACN), and the digested peptides were dried by a SpeedVac concentrator (Savant; Holbrook, NY). The peptides were finally resuspended in 0.1% formic acid prior to MS/MS analysis.

### 2.4 Analyses of proteins by nanoflow liquid chromatography coupled to tandem mass spectrometry (nanoLC-ESI-Qq-TOF MS/MS)

Separation of the digested peptides was performed using EASY-nLC II (Bruker Daltonics; Bremen, Germany). Briefly, peptides were loaded from a cooled (7 °C) autosampler into an in-house, 3-cm-long pre-column containing 5-μm C18 resin (Dr. Maisch GmbH; Ammerbuch, Germany) and then to an in-house, 10-cm-long analytical column packed with 3-μm C18 resin (Dr. Maisch GmbH) using mobile phase A (0.1% formic acid). The peptides were then separated by mobile phase B (ACN/0.1% formic acid) gradient elution (3–35%) for 150 min at a flow rate of 300 nl/min. Peptide sequences were then analyzed by an ultra-high resolution Qq-TOF MS/MS system (maXis Impact, Bruker Daltonics) in positive mode with ESI nanosprayer ion source. The nanoLC and Qq-TOF MS/MS systems were controlled by HyStar version 3.2 (Bruker Daltonics) and otofControl version 4.1 (Bruker Daltonics), respectively. A capillary voltage and spray shield voltage were set at 5,000V and 500 V, respectively. Nebulizer gas was set at 5.0 psi and dry gas flow rate was at 4.0 l/min, 150 °C [4], [5].

For MS scanning, precursor ions were scanned from 50 to 2200 *m/z* range (resolution = 40,000 at 622 *m/z*) and acquired at 2 Hz (0.5 s total accumulation). For MS/MS experiment, the three most intense precursor ions for every MS scan were selected for further fragmentation. Collision-induced dissociation (CID) MS/MS acquisition was performed at 2 Hz (0.5 s total accumulation, if precursor ≤ 1 × 10^4^ ion counts) and 10 Hz (0.1 s total accumulation, if precursor ≥ 5 × 10^5^ ion counts) on the same mass range and resolution set for MS scanning, whereas singly charged ions were excluded. Smart exclusion parameters were set to minimize repeated acquisitions of the same intense precursor ions (repeated count was 2, dynamic exclusion was set at 0.50 min).

### 2.5 MS/MS data processing and protein identification

The raw files (.d) were charge-deconvoluted and extracted into peak list files (.mgf) using Data Analysis version 4.1 software (Bruker Daltonics) via an embedded daMGF script. For identification, the peak list files (.mgf) were searched against the human Swiss-Prot database using Mascot 2.4 search engine (Matrix Science; London, UK). Fixed modification was carbamidomethylation at cysteine residues, whereas variable modification was oxidation at methionine residues. Enzyme was specified to trypsin and only one missed cleavage per peptide was allowed. Data searches were performed with a precursor tolerance of 0.1 Da and fragmentation tolerance of 0.5 Da with +2 and +3 charge state. The false discovery rate (FDR) was performed by searching the decoy database and adjusted to <1% at protein level [5], [6].

### 2.6 Quantitative analysis of MS/MS spectra

Intensity analysis of MS/MS spectra derived from each identified protein was performed using Skyline v.3.5 software (http://proteome.gs.washington.edu/software/skyline). Briefly, The Mascot search result files (.dat) were imported to generate the spectral libraries using BiblioSpec algorithm by the following parameters: spectra cut-off score = 0.95, peptide length = 8–25 amino acids, and share peptides assigned for particular protein. Subsequently, raw files (.d) were then imported and matched to validated peptides in spectral libraries to generate precursor peptide ion intensity. These precursor peptide ion intensities represented the summation of an area under curve of extracted ion chromatograms (XICs) containing three precursor ion isotope peaks (M, M+1, and M+2) [5]. Finally, the total ion intensity of precursor peptide assigned for a given protein was exported and is reported for individual biological replicates, whereas the quantitative intensity data for peptide MS/MS spectra that did not meet the aforementioned predefined criteria are reported as “not applicable”.
